# De novo genome assembly of *Oryza granulata* reveals rapid genome expansion and adaptive evolution

**DOI:** 10.1038/s42003-018-0089-4

**Published:** 2018-06-29

**Authors:** Zhigang Wu, Dongming Fang, Rui Yang, Fei Gao, Xingyu An, Xiaoxuan Zhuo, Yafei Li, Chuandeng Yi, Tao Zhang, Chengzhi Liang, Peng Cui, Zhukuan Cheng, Qiong Luo

**Affiliations:** 1 State Key Laboratory for Conservation and Utilization of Bio-Resources in Yunnan, Ministry of Education Key Laboratory of Agriculture Biodiversity for Plant Disease Management, Yunnan Agricultural University, 650201 Kunming, China; 2 Agricultural Genomics Institute at Shenzhen, Chinese Academy of Agricultural Sciences, 518120 Shenzhen, Guangdong China; 3 State Key Laboratory of Plant Genomics, Institute of Genetics and Developmental Biology, Chinese Academy of Sciences, 100101 Beijing, China; 4 Agricultural College, Yangzhou University, 225009 Yangzhou, China

## Abstract

The wild relatives of rice have adapted to different ecological environments and constitute a useful reservoir of agronomic traits for genetic improvement. Here we present the ~777 Mb de novo assembled genome sequence of *Oryza granulata*. Recent bursts of long-terminal repeat retrotransposons, especially *RIRE2*, led to a rapid twofold increase in genome size after *O. granulata* speciation. Universal centromeric tandem repeats are absent within its centromeres, while *gypsy*-type LTRs constitute the main centromere-specific repetitive elements. A total of 40,116 protein-coding genes were predicted in *O. granulata*, which is close to that of *Oryza sativa*. Both the copy number and function of genes involved in photosynthesis and energy production have undergone positive selection during the evolution of *O. granulata*, which might have facilitated its adaptation to the low light habitats. Together, our findings reveal the rapid genome expansion, distinctive centromere organization, and adaptive evolution of *O. granulata*.

## Abstract (web summary)

Zhigang Wu, Dongming Fang, Rui Yang, et al. present the genome assembly of a wild rice species *Oryza granulata*, revealing critical insights about the rapid genome expansion and evolution observed in the *Oryza* genus. They find that recent bursts of LTR retrotransposons have led to the rapid increase in *O. granulate* genome size following speciation.

## Introduction

Rapid advancements in sequencing technology have facilitated comparative genomics based on fully sequenced genomes for studying gene and genome evolution^1^. In plants, whole-genome duplication, sequence reorganization, and transposable element (TE) proliferation have complicated genome organization greatly^2^. Even within closely related species, such as those in the genus *Oryza* or *Arabidopsis*, remarkable fluctuation in gene collinearity and genome size has been observed^3–5^. Comparative analysis of orthologous regions or whole-genomic sequences within closely related species, especially within the same genus, may shed more light on the mechanism of genome evolution.

The genus *Oryza* contains 24 species, including two cultivated and 22 wild species, distributed throughout the world. Based on morphological, cytological, and molecular divergence studies, they have been classified into ten genome types, namely AA, BB, CC, BBCC, CCDD, EE, FF, GG, HHJJ, and HHKK^6,7^. The two cultivated species, *O. sativa* (Asian cultivar) and *Oryza glaberrima* (African cultivar) both with AA genome, have been extensively sequenced and studied in the past years, because of their outstanding contribution to world food security^8–10^. The wild rice genomes not only constitute a useful reservoir of genetic diversity, contributing important genetic resources for rice breeding^11^, but also provide fundamental data for studying plant genome evolution within a short timeframe. However, only two types of wild rice genomes, the AA and FF genomes, have been published thus far^4,12^. The wild rice *O. granulata* possesses a GG genome and has been placed within the basal lineage of the genus *Oryza* with the second largest genome among the diploid rice species^7,13^. Its genome is dramatically expanded, with a genome size twice that of *O. sativa*, and contains a different set of repeat sequences relative to other *Oryza* species^14,15^. Furthermore, *O. granulata* also has several distinctive natural traits, as it is photoperiod-insensitive and tolerant to shade^16^. Therefore, comparative analyses of *O. granulata* with other sequenced *Oryza* genomes provide a unique opportunity to explore genomic size variation and adaptive evolution in this genus.

Centromeres are chromosomal sites for kinetochore assembly and spindle attachment, thereby enabling accurate chromosome segregation during each cell cycle. The presence of CENH3, a centromere-specific histone H3 variant, is the hallmark of all functional centromeres^17^. In most animals and plants that have been studied, centromeres are composed of long arrays of satellite repeats and/or long-terminal repeats (LTRs)^18,19^. Centromere functions are conserved among all eukaryotes, yet their sequences evolve rapidly and show tremendous variation both in size and organization pattern, even between closely related species and different chromosomes^19,20^. Cultivated rice and its wild relatives provide an excellent system for exploring centromeric sequences evolution^21^. Centromeres of *O. sativa* are mainly composed of a 155-bp satellite repeat CentO and CRR (Centromeric Retrotransposon of Rice) elements, which both are closely intermingled^22^. CRR is derived from a *Ty3/gypsy* class of retrotransposon and the amount of CentO in each centromere varies from ~60 kb to ~2 Mb. A recent study reported that *Gran3* retrotransposons are the functional centromere elements of *O. granulata*; however, FISH signals of *Gran3* are not restricted to centromeric regions but dispersed across both the centromeric and pericentromeric regions^23^. Thus, the centromere-specific elements of *O. granulata* remain enigmatic.

In this study, we use single-molecule real-time (SMRT) sequencing approach combined with a fosmid library to assemble ~777 Mb of the *O. granulata* genome. *O. granulata* has an expanded genome, nearly double that of *O. sativa*, composed of more than 67% of repeat elements. We show that recent bursting of LTRs, especially *RIRE2*, *ATLANTYS*, and *Copia*, contribute greatly to the rapid expansion of the *O. granulata* genome. No tandem repeats were screened out in the centromeric sequence library, demonstrating that the centromeres of *O. granulata* are lack of tandem repeats. We annotated 40,116 genes in *O. granulata*, which is comparable to that of *O. sativa*^13^. Both the copy number and function of genes involved in photosynthesis and energy production have undergone positive selection, which would facilitate the adaptation of *O. granulata* to its low light habitats.

## Results

### De novo assembly and annotation of the *O. granulata* genome

We sequenced *O. granulata* genomic DNA to generate 20.58 Gb (~26×) pair-end (2 × 150 bp), 79.51 Gb (~102×) pair-end (2 × 250 bp) reads using Illumina GA II platform, and 16.6 Gb (~21×) PacBio single-molecule long reads (with 8.7 Kb average length) from 15 cells using P5 polymerase binding and C3 chemistry (Supplementary Table 1). We also sequenced 384 fosmid pools, each containing ∼ 1000 independent clones with insert size of ∼40 kb, to produce 5.06 Gb of GBS tags of length 2 × 150 bp (Supplementary Table 1).

We performed a PacBio-only assembly using an overlap-layout-consensus method implemented in Canu^24^. Due to the small size of DNA libraries, short read lengths of Illumina sequencing, and high level of repeat sequences, the Illumina-only assembly was highly fragmented and not complete using four other assembly methods: SOAPdenovo2^25^, BASE, DiscoverDeNovo, and MaSuRCA. Based on contig N50 results, SOAPdenovo2 long nonredundant contigs were selected and added to the PacBio-only assembly. Subsequently, Illumina short reads were mapped onto the assembly using BWA (keeping only unique alignments with mapping quality >20). Then, GATK was used to call variants, including SNPs and indels. We corrected homozygous SNPs and indels with at least five supporting reads. Finally, the custom tool Pilon^26^ was used to polish the assembly and correct remaining indel errors. The final assembled sequence was 776.96 Mb with a contig N50 size of 262 kb (Supplementary Table 2), which is nearly twice that of *O. sativa*, and by far the largest of the published *Oryza* genomes. The genome size is consistent with *K*-mer analysis from short reads estimated at ~785 Mb (Supplementary Fig. 1 and Supplementary Table 3), and a previous study by flow cytometry estimated at 882 Mb^13^.

To assess the coverage and completeness of the genome assembly, we generated low-coverage (~6×) mate-pair reads from a ~40 kb fosmid library. Most (~98.90%) of the mate-pair reads were unambiguously mapped onto the final assembly (Supplementary Table 1), exhibiting a peak at ~34 kb (~23.84%) mapping distance (Supplementary Fig. 2). In addition, using RNA sequencing data generated from the root, stem, sheath, panicle, and leaf tissues of *O. granulata*, 92.70–93.46% of the reads could be mapped onto the final assembled sequence (Supplementary Table 4). Additional quality analysis indicated that 95.16% of the core eukaryotic genes (CEGMA) were present in our *O. granulata* assembly^27^, 97.98% were partially represented (Supplementary Table 2). For further assessment of the assembly and annotation completeness, we used the software tool BUSCO (Benchmarking Universal Single-Copy Orthologs), which indicated that 95% of the set consists of complete single-copy BUSCOs^28^ (Supplementary Table 2), suggesting near completeness of euchromatin components. Taken together, these results indicate that our genome assembly is of high quality.

By combining ab initio RNA-seq reads and plant genes and domains, we predicted that the *O. granulata* genome contains 40,116 protein-coding genes (Supplementary Table 2), of which 84.05% are supported by at least ten RNA-seq reads obtained from five *O. granulata* tissues (Supplementary Fig. 3). Moreover, of the inferred proteins, 33,901 match entries in the SWISS-PROT, InterPro, KEGG, or TrEMBL databases (Supplementary Table 5). We also annotated 2038 ncRNAs, including 1083 tRNAs, 722 rRNAs, and 233 microRNAs (miRNAs) encoding genes (Supplementary Table 6).

### Repeat sequences in *O. granulata*

Approximately 67.96% (~528.04 Mb) of the *O. granulata* genome is composed of repetitive sequences, which is much higher than that determined for *O. sativa* (~45.52%)^8^, indicating that expansion of repeat sequences occurred in *O. granulata* (Supplementary Table 7). As a result, *O. granulata* has 358.12 Mb more repetitive sequences than *O. sativa*, which is the major determinant of genome expansion in *O. granulata*. Comparative analysis of high-frequency *K*-mers from the short reads of the *O. granulata* and *O. sativa* genomes indicated that there are more specific *K*-mers in the *O. granulata* genome relative to *O. sativa*. However, very few common *K*-mers were identified (Supplementary Table 8), suggesting that there is a large difference in the composition of repeat sequences between the two genomes. These results indicate that repeat sequences in the *O. granulata* genome evolved in a distinctive way in the genus *Oryza*.

Retrotransposons, especially long-terminal repeat (LTR) retrotransposons, are the most abundant repeat elements in *O. granulata*, accounting for ~59.33% of the *O. granulata* genome (Supplementary Table 9). Aside from LTRs, annotated tandem repeats and DNA transposons represent only 2.42 and 8.80% of the genome, respectively (Supplementary Tables 7 and 9). A total of 250 LTR retrotransposon families (masked size >500 Kb) were identified in *O. granulata*, including 39 and 200 members of the *Copia* and *Gypsy* families, respectively. *Gypsy*-type LTRs, one of the dominant forms of repeat elements found in plant genomes, are the most abundant subtype present in *O. granulata*, accounting for ~407.04 Mb of the genome (Supplementary Table 10). In addition to *Gypsy*-type LTRs, *Copia*-type LTRs account for ~54.90 Mb of the genome (Supplementary Table 10). In contrast, *Gypsy*- and *Copia*-type LTRs are only comprised 83.48 and 20.10 Mb in *O. sativa*, respectively (Supplementary Table 10). These data indicate that *Gypsy*-type LTRs are five times more abundant in *O. granulata* relative to *O. sativa*, suggesting that bursts of *Gypsy*-type LTRs might be responsible for the increased genome size observed in *O. granulata*.

Further analysis of *Gypsy*- and *Copia*-type LTR families indicated that three subtypes, namely *RIRE2*, *ATLANTYS*, and *Copia*, are significantly expanded in *O. granulata* as compared with those of *O. sativa* (*P* < 0.01). Furthermore, *RIRE2*, *ATLANTYS*, and *Copia* subtypes make up approximately 133.18 Mb (i.e. 17.14%), 48.16 Mb (i.e. 6.20%), and 47.35 Mb (i.e. 6.09%), respectively, of the genome of *O. granulata* when considered separately (Supplementary Tables 11 and 12). In contrast, these three LTR subtypes only contribute 5.97 Mb (i.e. 1.60%), 8.68 Mb (i.e. 2.33%), and 10.85 Mb (i.e. 2.91%), respectively, to the *O. sativa* genome, suggesting that the amplification of these typical LTRs, especially *RIRE2*, plays a dominant role in the expansion of repeat sequences in *O. granulata*.

**Fig. 1:**
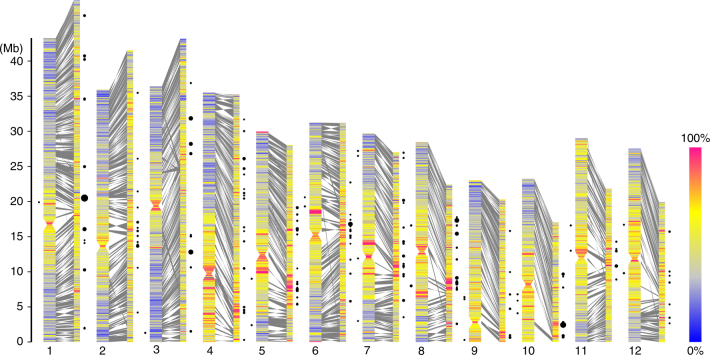
Characterization of 1597 collinear gene blocks between *O. granulata* and *O. sativa* genome. Chromosome plots of the *O. sativa* genome (left), comprising 1019 contigs with collinear gene blocks of *O. granulata* (right) that cover ~45.86 % of the assembly size. Chromosomes and contigs filled with density of repeat elements for every 100 kb window. Syntenic regions are shaded in gray boxes between them. Black circles show the percentage masked by *RIRE2* in a window, and only where the proportion is more than 0.2 here

The *RIRE2*-LTR is a 441 bp DNA segment, which was first identified as a Gypsy-type retrotransposon in rice^29^. Typically, *RIRE2* has a long internal region that is almost 10 kb long (Supplementary Fig. 4), which contains two long open reading frames (ORF1 and ORF2) encoding the *gag*, *pro*, *rt*, *rh*, and *int* genes in that order. In the *O. granulata* genome, 70% of *RIRE2-*LTRs have intact ORFs, and the remaining 30% have been partially deleted, which can be illustrated by plotting the coverage of each ORF along each *RIRE2* sequence. Moreover, by plotting the positions of *RIRE2* insertions and comparing them to those of the *O. sativa* genome, we found that *RIRE2* is frequently inserted into the regions surrounding centromeres (Fig. 1).

### Evolutionary history of repeat sequences in *O. granulata*

Our data indicated that *RIRE2*, *ATLANTYS*, and *Copia* LTRs are remarkably expanded in *O. granulata*, contributing a total of 228 Mb to the genome. By homology searching with sequences of *RIRE2*, *ATLANTYS*, and *Copia* LTRs, we found that all the three LTRs are also present in *Oryza brachyantha* and *O. sativa* (Supplementary Tables 11 and 12), suggesting an ancient origin of these LTRs in the genus *Oryza*. In order to characterize and date the transposition burst of these elements in *O. granulata*, we conducted phylogenetic analyses of the complete element conservation (supported by conserved region more than 90% coverage) in the three *Oryza* species (Supplementary Fig. 5). Our data showed that paralogs of the elements found in each *Oryza* species form a cluster, which is distinct from those found in the other two *Oryza* species, suggesting that the retrotransposition bursts either occurred concomitantly or after the speciation of *O. granulata*.

**Fig. 2:**
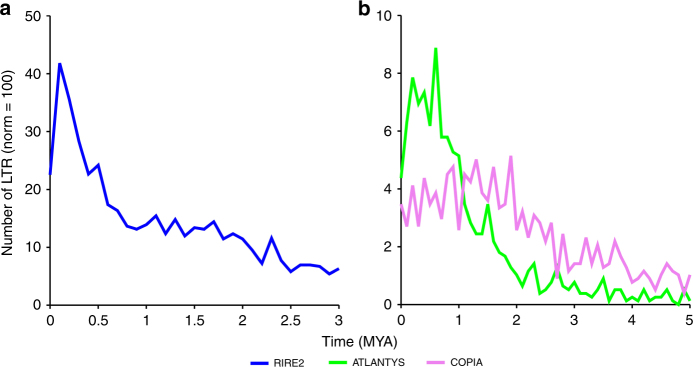
Timing of the bursts of the three retrotransposons, *RIRE2*, *ATLANTYS*, and *COPIA*, in *O. granulata*. **a** Timing of the burst of *RIRE2*. **b** Timing of the burst of *ATLANTYS* and *COPIA*. For each LTR retrotransposons in *O. granulata* genome, the curves represent the date of divergence in MYA translated from the observed divergence, using the molecular clock (1.3×10^−^^8^ substitutions per site per year from Ma and Bennetzen^47^). The groups of paralogs used to compute the pairwise distances are defined with EMBOSS. The *y*-axis represents the total number of copy equivalents

In order to test this hypothesis, we estimated the dates of retrotransposition of these three LTR subtypes in the *O. granulata* genome. In the case of full-length LTR retrotransposons, the date of insertion can be estimated based on the divergence between these two LTRs. The results showed that these LTRs have different transpositional activities. *RIRE2* showed a recent burst at 0.1–0.4 MYA (Fig. 2a), while *ATLANTYS* and *Copia* showed relatively earlier bursts at 0.5–0.8 and 1.0–2.0 MYA, respectively (Fig. 2b). Our data indicated that these elements expanded more recently, and occurred much later than the divergence date of *O. granulata* species. Thus, we propose that this repeat-driven genomic expansion is posterior to and not concomitant with speciation.

### GG genome centromeres lack tandem repeats

**Fig. 3:**
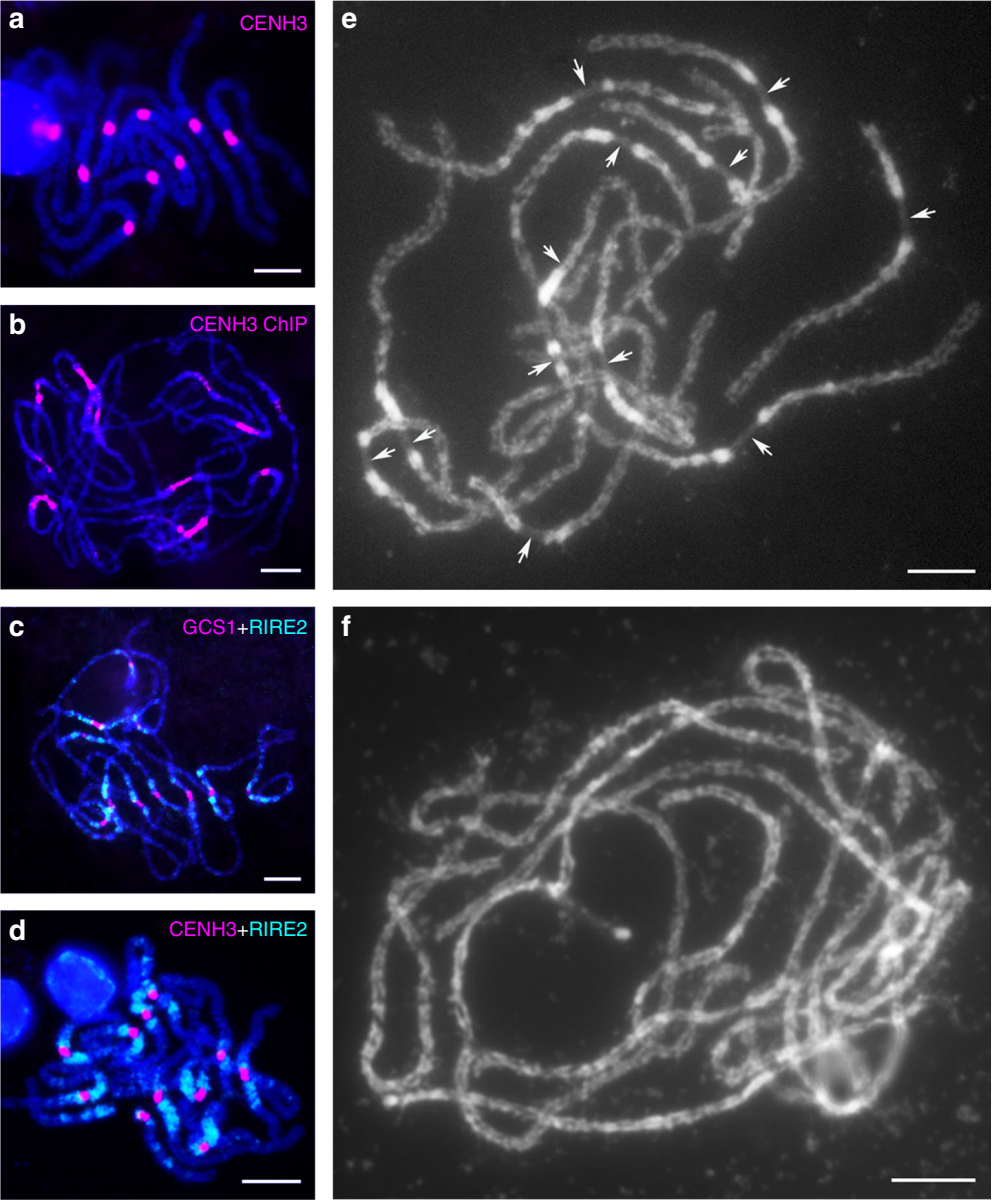
FISH mapping of centromeric and pericentromeric probes on pachytene chromosomes of *O. granulata* and phenotypic characterization of its pachytene chromosomes. **a** Immunodetection of rabbit antibody against rice CENH3 on pachytene chromosomes of *O. granulata*. **b** FISH identification of precipitated DNA isolated by ChIP using rice anti-CENH3 antibodies. **c** Dual color FISH of GCS1 cloned from ChIP DNAs and *RIRE2*. GCS1 was labeled with digoxigenin-dUTP (magenta), while *RIRE2* was labeled with biotin-dUTP (cyan). **d** Immuno-FISH detection of *RIRE2*. Rice anti-CENH3 antibodies were used to label centromeres. **e** Pachytene chromosome morphology of *O. granulata*. Arrows indicate the 12 centromere constrictions on each pachytene chromosome of *O. granulata*. **f** Pachytene chromosome morphology of *O. sativa*. Chromosomes were counterstained with DAPI. Scale bars = 5 μm

Centromeres usually contain an abundance of repetitive sequences, including tandem repeats and retrotransposons. However, tandem repeats were not found in centromeric regions of *O. granulata* chromosomes by FISH assays. In order to identify the centromeric elements of *O. granulata*, ChIP cloning was carried out using a rice anti-CENH3 antibody that showed high specificity to *O. granulata* centromeres (Fig. 3a). Using the ChIP DNAs as FISH probes, we found that they are highly enriched in centromeric regions of *O. granulata* chromosomes (Fig. 3b). In order to obtain more details of the centromeric elements in *O. granulata*, a plasmid library of ChIP DNAs was generated, and 34 clones with an insert size larger than 1000 bp were randomly selected as FISH probes. Probes with positive FISH signals in the centromeric regions of *O. granulata* chromosomes were perceived as Granulata Centromere Sequence (GCS). GCS1 was the first identified probe that showed unambiguous magenta FISH signals in the centromeric regions of 12 *O. granulata* chromosomes, flanked by obvious cyan FISH signals of pericentromeric probe *RIRE2* (Fig. 3c). We also identified additional four GCSs, including GCS2, GCS3, GCS4, and GCS5, were localized in the centromeric regions of *O. granulata* chromosomes (Supplementary Fig. 6). Nevertheless, Immuno-FISH experiments confirmed that *RIRE2* was mainly located in pericentromeric regions but not in centromeric regions of *O. granulata* chromosomes (Fig. 3d).

Next, a total of 65 contigs (Supplementary Table 13) were screened out by BLAST searching analysis of the assembled genome using the sequences of GCS1−GCS5, resulting in the identification of a 7.36 Mb sequence library proposed to represent the centromeric DNA of *O. granulata*. About 45% of this library is composed of interspersed repeats, in which *gypsy*-type LTRs accounted for about 81.37%; whereas no tandem repeats were found in this library (Supplementary Table 14), which further confirmed the centromeres of *O. granulata* are lacking tandem repeats. Further sequence analysis revealed that the Centromeric Retrotransposon of Maize (CRM) family is the major centromeric LTR sequence present in *O. granulata* (Supplementary Table 15). Analysis of centromeric contig2720 using LTR_FINDER^30^ showed that centromere-specific LTRs of *O. granulata* are inserted into each other, and abundant LTR insert traces were found within the full-length LTRs (Supplementary Fig. 7 and Supplementary Data 1). Collectively, centromeres of *O. granulata* chromosomes lack tandem repeats and are primarily composed of unique sequences that are interspersed with *gypsy*-type LTRs.

4′,6-diamidino-2-phenylindole (DAPI)-stained pachytene chromosomes were further investigated to understand the characteristics of centromeres that are naturally lacking tandem repetitive sequences in *O. granulata*. We found that the centromeric region of each *O. granulata* pachytene chromosome was distinctly constricted (Fig. 3e). This phenomenon was never observed in *O. sativa* (Fig. 3f) or in other species of the genus *Oryza* (Supplementary Fig. 8).

### Repeat-driven evolution of intron size in *O. granulata*

Pairwise comparisons of exons and introns of homologous genes detection as Reciprocal Best Hits indicated that exon length is conserved among *Oryza* species, while intron length varied statistically significantly (*t* test in R package, *P* value = 7.325e-16). The average length of individual introns was found to be 378.87 bp and 415.94 bp for *O. sativa* and *O. granulata*, respectively (Supplementary Table 16 and Supplementary Fig. 9). Taken together, these data suggest that intron length increased as *O. granulata* evolved over time.

Next, we investigated whether the presence of more repetitive sequences may represent a possible origin of the long introns observed in *O. granulata*. The results showed that the expanded *O. granulata* introns identified in our study, relative to their homologous counterparts in other *Oryza* species, contain TEs (Supplementary Fig. 10), indicating that insertion of TEs is the key factor driving the increase of intron size in *O. granulata* (cor.test in R package, *R* = 0.95). Sequence analysis also revealed that the largest TE insertion was classified as a *RIRE2* LTR (Supplementary Table 17), and the expansion of *RIRE2* LTRs may specifically contribute to the observed increased of intron lengths. These data suggest that in *O. granulata*, gene length should be increased as consequence of intron expansion. Indeed, comparative analysis of length of homologous genes between *O. granulata* and *O. sativa* showed that *O. granulata* has, on average, longer genes than *O. sativa* (3.27 vs. 3.06Kb) (Supplementary Data 1 and Supplementary Fig. 11). Taken together, we propose that the expansion of TEs in the intronic regions of the *O. granulata* genome directly led to the observed increased gene size.

### Gene family evolution in *O. granulata*

**Fig. 4:**
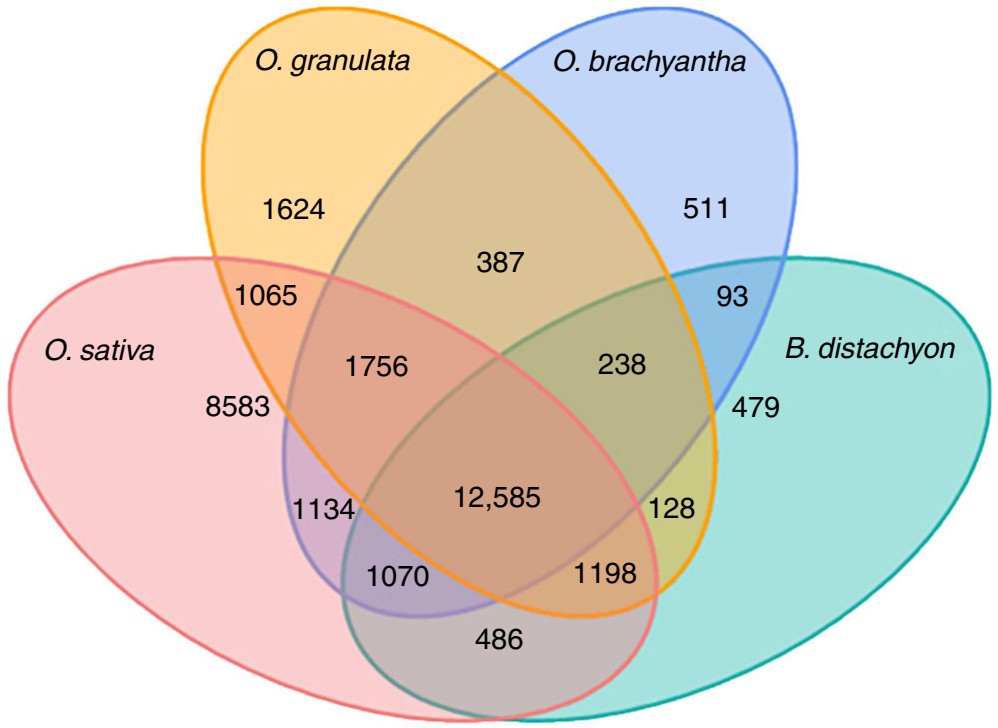
Venn diagram showing the distribution of gene families between *O. granulata*, *O. brachyantha*, *O. sativa*, and *B. distachyon*. The intersections between species indicate the numbers of shared gene families, while unique family numbers are shown in species-specific areas. The center represents the number of families shared by all species simultaneously. Only families with four or more protein members were considered. Analysis of families showed 12,585 families shared by all species simultaneously

We identified 18,981 gene families in *O. granulata* based on predicted protein-coding genes. More than 90% of the identified gene families (17,229) are shared by both *O. sativa* and/or *O. brachyantha* (Fig. 4). Interestingly, we identified 1752 gene families that are only present in *O. granulata*, but are absent in both *O. sativa* and *O. brachyantha*. Analysis of GO terms and InterPro domains for these lineage-specific families revealed that several functional pathways involved in DNA integration, photosynthesis, protein translation, and ATP biosynthesis are enriched in *O. granulata* (Supplementary Table 18).

Expansion or contraction of gene families is often associated with the adaptations of plants to different environoments^31^. As compared with both of *O. sativa* and *O. brachyantha*, 420 gene families with a bigger size were expanded in *O. granulata*, whereas only two of them were found containing a few members (the size of family required at least five genes in *O. granulata*, and the number of genes from one family required twofold changes). GO and InterPro domain analyses of the expanded families indicated that those protein families associated with TEs, such as the transposase-derived protein, ribonuclease H-like domain, Aminotransferase-like, and plant mobile domain families are extremely overrepresented in *O. granulata* (Supplementary Table 19 and Supplementary Data 2).

Gene families associated with photosynthesis and ATP synthesis are also obviously expanded in *O. granulata* (Supplementary Data 3 and 4). We identified 154 genes encoding the components of photosystem I and II in *O. granulata*, in contrast to the presence of only 40 genes in *O. sativa* and 7 in *O. brachyantha*, respectively. We also identified 68 genes encoding ATPase, which is apparently much more than that 16 and 6 genes present in *O. sativa* and *O. brachyantha*, respectively. This expansion suggests that there has been stronger selection for enhanced light utilization and efficiency of energy production in *O. granulata*. This positive selection is consistent with the shade-requiring growth habit of *O. granulata*. In comparison with other *Oryza* species, *O. granulata* usually grows in well-drained soil under trees on small hills, and is therefore often shaded during peak daylight hours. Therefore, the expansion of these genes may play important roles in the adaptation to the lower light environment.

### Positive selection of *O. granulata* genes

**Fig. 5:**
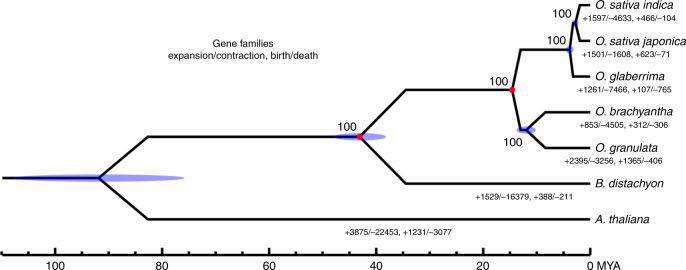
Phylogeny and divergence time estimation by molecular clock analysis. Divergence times of seven *Oryza* species were calculated using the first codon positions of 3746 single-copy protein-coding genes. Tree topology is supported by 100% bootstrap values. The blue ellipses on the nodes indicate the 95% credibility intervals of the estimated posterior distributions of the divergence times. The red circles indicate the fossil calibration times used for setting the upper and lower bounds of the estimates. MYA million years ago

The seven closely related rice genomes provide a phylogenetic framework to examine protein-coding genes showing accelerated molecular evolution in rice species and detect signals of positive selection on genes involved in adaptive divergence (Fig. 5). We identified 3746 single-copy gene families displaying accelerated evolution (Supplementary Table 20). GO/IPR classification (Supplementary Tables 21 and 22) showed that the detected positive selection genes (PSGs) spanned a wide range of functional categories. Notably, there is a statistically significant (*P* < 0.05) enrichment of 171 PSGs involved in pathways related to energy synthesis and metabolism (Supplementary Data 5 and 6). Of these 171 PSGs, 30 genes involved in energy synthesis and metabolism categories show evidence for positive selection. These results suggested that the function of the genes involved in photosynthesis and energy production underwent positive selection during the evolution of *O. granulata*.

In addition, ten genes involved in protein translation are PSGs. Of these genes, five are associated with tRNA biosynthesis and two are involved in transcriptional elongation, suggesting the enhanced selection for translation in *O. granulata*. Although the direct reason behind an expansion of gene families involved in protein synthesis remains unclear, we speculate the large expansions observed in families related to photosynthesis in *O. granulata* may contribute a lot to this expansion.

## Discussion

The genus *Oryza* represents an excellent system for studying genomic evolution in plants. In addition to the two cultivated rice species *O. sativa* and *O. glaberrima*, the reference genomes of wild species with AA and FF genomes have been released^4,12^. *O. granulata* is one of the oldest wild rice species in the genus *Oryza*^9^, and its GG type genome shows obvious distinctions from both the AA and FF genomes^13^. Moreover, *O. granulata* is the only wild *Oryza* species that grows in upland habitats not in or near water. Therefore, *O. granulata* provides an excellent opportunity to explore *Oryza* genome evolution.

Here we present a ~780 Mb genome of the wild rice, *O. granulata*, using SMRT sequencing. The genome is about twice the size of *O. sativa*, which is the largest genome among the sequenced *Oryza* species so far. In plants, with the exception of polyploidization, TE proliferation is a primary mechanism leading to genome expansion^2,32^. Similarly, we showed that the expansion of the *O. granulata* genome also resulted from insertions of LTR retrotransposons. We also revealed that three subtypes of LTR families, *RIRE2*, *ATLANTYS*, and *Copia*, expanded rapidly in *O. granulata*, making a large contribution to its expanded genome size. Homologs of these three elements were found in nearly all the published *Oryza* genomes, suggesting that these elements were present in the common ancestor of the *Oryza* genus. Examination of complete sequences of these LTR retrotransposons revealed that they had undergone independent amplification around 1–3 million years ago, after the speciation of *O. granulata*. Furthermore, FISH identification analyses revealed that *RIRE2* is mainly inserted into pericentromeric regions of *O. granulata* chromosomes. Those results provide direct evidence that the activity of LTR retrotransposons may increase genome size to such a degree over a short evolutionary period.

TE amplification is a nonnegligible driving force for genome expansion and gene collinearity erosion^1,3^. We provided direct evidence that bursting of *Gypsy*-type LTRs is responsible for the GG genome expansion. In addition, we conducted statistical analysis of collinearity between *O. sativa* and *O. granulata*, and other four species, 1597 *O. granulata* vs. *O. sativa* syntenic gene blocks were identified, which are only comprised of 30.15% (16,875) of *O. sativa* genes (Supplementary Tables 23 and 24). We also detected fewer collinear blocks between *O. granulata* and *O. nivara*, or between *O. granulata* and *O. brachyantha* (Supplementary Fig. 12). While in the noncollinear regions, repetitive sequences are more prevalent than collinear blocks (Supplementary Figs. 13 and 14). These results suggest repetitive sequences might integration into noncollinear genes in *O. granulata*, leading to the erosion of gene collinearity. However, due to the lack of chromosome level assembly, more detailed analysis of gene collinearity remains to be performed.

Centromere-specific satellite repeats are intrinsic for their biological functions. In humans, alpha satellites are sufficient for artificial chromosome constructions, while neocentromeric DNA is incompetent^33^. Centromere-specific satellites are the most common feature of centromeres reported so far^18,19^. However, some extreme and/or exceptional cases also exist. For example, the rice Cen8 contains only ~65 kb of the centromeric repeat CentO, accounting less than 10% of the functional centromere^34^. Five potato centromeres (Cen4, Cen6, Cen10, Cen11, and Cen12) lack satellite repeats and only consist of single- or low-copy DNA sequences^20^. Unexpected, we could not detect any tandem repeats at centromere regions of *O. granulata*, only with LTR retrotransposons. It is the single genome without any satellite repeats at centromeric regions identified in the genus *Oryza* so far. However, in the wild rice *Oryza punctata* (BB), a 165 bp CentO, containing a 10 bp insertion (TTTATAGGCA) compared with the 155 bp monomer of *O. sativa*, is the major component of its functional centromeres^35^. ChIP cloning using antibodies against CENH3 also reveal that satellite repeats, including the 126 bp CentO-C1, 366 bp CentO-C2, and 154 bp CentO-F, are genome-specific centromeric elements of *O. rhizomatis* (CC) and *O. brachyantha* (FF), respectively^14^. Southern blot hybridization using CentO, CentO-C1, CentO-C2, and CentO-F on digested genomic DNA of *O. granulata* suggests that CentO repeats have either diverged substantially or have been replaced by unrelated sequences^14^.

The presence of two extreme types of centromeres in the *Oryza* genus supports the hypothesis of a sudden, rather than gradual, evolutionary transition from tandem-repeatless to satellite repeat-based centromeres, similar to the phenomenon observed in potato^22^. Amplified DNA from retrotransposons appears to be a major source for the generation of new centromeric satellite repeats^36,37^. There are abundant LTR retrotransposons inserted into each other frequently in the centromeres of GG genome. The high-frequency insertion of centromere-specific LTRs in GG genome would make some centromeres bigger than their homologous chromosomes. According to meiotic drive hypothesis, haploid gametes with a large centromeric region will increase their transmission to the egg^38^. Thus we suspect that the GG genome is still on the way towards generating centromere-specific satellite repeats. In addition, we found the CRM families, centromere-specific LTRs of maize^39^, are the most prominent components of the centromere-specific LTRs in the GG genome, suggesting that CRM families might be reserved centromeric sequences from their common ancestor. Collectively, centromere organization in the GG genome provides direct evidence that centromeres lacking tandem repeats may also stably exist and CR elements might be a key driving force during the evolution.

The expansion of gene families and accelerated molecular evolutionary rates are often associated with positive natural selection and adaptive evolution in plants. In *O. granulata*, we detected several protein-coding genes under considerable positive selection that are mainly involved in photosynthesis and energy production. These genes are thought to be responsible for adaptive evolution to the native shade-requiring environment of *O. granulata*. We also detected several other genes under the positive selection involved in translation pathways.

In summary, we generated a high-quality reference genome sequence of *O. granulata*. Comparative genomic analyses among *Oryza* genomes reveal possible mechanisms underlying genome size expansion, centromere evolution, and gene adaption, among other processes. Moreover, the gene family expansion and high evolutionary rate are well associated with the adaptation of *O. granulata* to its native environment.

## Methods

### Plant materials

Plants of *O. granulata* (IRGC Acc. No.102117) were kindly provided by the International Rice Research Institute (IRRI) and cultivated in greenhouse under appropriate shading conditions. Panicles of Zhongxian 3037 and wild rice species, including *O. punctata* (BB), *Oryza officinalis* (CC), *Oryza australiensis* (EE), and *O. brachyantha* (FF), were kept in Yangzhou University and used for cytological studies.

### Sequencing and assembly

Genomic DNA of *O. granulata* was extracted from young leaves with the DNeasy Plant Mini Kit (Qiagen, USA). Sequencing was performed with both the PacBio SMRT and Illumina Hiseq platforms. We constructed the fosmid library consisting of about 2.5 million clones using the CopyControl™ Fosmid Library Production Kit with pCC1FOS™ Vector (Epicentre, USA).

Total RNA from roots, stems, leaves, leaf sheaths, and panicles was isolated using the TRIzol^®^ Reagent (Thermo Fisher Scientific, USA). We then used Dynabeads with Oligo (dT) primers to enrich for mRNA. Enriched mRNA was fragmented into 200–700 nucleotide fragments and reverse-transcribed into cDNA using random hexamer primers. The products were purified with the QIAquick PCR Purification Kit, and ~250 bp fragments were used to construct the sequencing library using the mRNA-seq sample prep kit (Illumina). Sequencing was performed with the Illumina Genome Analyzer (150 bp PE).

The genome size of *O. granulata* was estimated by *K*-mer frequencies according to the Lander−Waterman theory^40^. The genome was initially assembled using Canu (1.0) with the PacBio reads^24^, according to the following four steps: (1) Detecting overlaps in high-noise sequences using MHAP^41^; (2) Generating corrected sequence consensus; (3) Trimming corrected sequences, including suspicious regions, such as remaining SMRTbell adapters; and (4) Assembling trimmed corrected sequences. For Canu, the following parameters were used: errorRate = 0.01 and genomeSize = 780 Mb. Default parameters were otherwise employed for Canu assembly. We also tested other assemblers to estimate the PacBio Canu assembly results. Raw Illumina HiSeq data from three libraries of small insert sizes (<800 bp) were assembled by SOAPdenovo2 with different *K*-mer size^25^. Moreover, WGS Illumina sequences from *O. granulata* DNA were used to assess the error rate of the integrated assembly and residual within-genome heterozygosity. High-quality data were aligned to the assembly using BWA mem (0.7.10)^42^, followed by application of GATK (3.2-2) to call variants with a minimum coverage threshold of ten reads^43^, including SNPs and indels. We corrected homozygous SNPs and indels with at least five supporting reads. Finally, these reads were used to polish the assembly using Pilon^26^ (https://github.com/broadinstitute/pilon), a custom tool to correct remaining indel errors.

### Repeat annotation

Repeat sequences in the *O. granulata* genome were identified using the following procedures. First, tandem repeats were identified across the genome using Tandem Repeats Finder software (4.07)^44^. Next, TEs were predicted by homology-based methods using RepeatMasker (4.0.6) (http://www.repeatmasker.org/) and RepeatProteinMask, and de novo approaches using LTR_STRUCT^45^, LTR_FINDER (1.0.6)^30^, and RepeatScout (1.0.5)^46^. RepeatMasker was employed for DNA-level identification using a general library (RepBase20.04). At the protein level, RepeatProteinMask (the updated software as included in the RepeatMasker package) was employed for a WuBlastX searching against the TE protein database. LTR_STRUCT and LTR_FINDER were used to search the whole genome for characterizing structures of full-length LTR retrotransposons. RepeatScout built consensus sequences using fit-preferred alignment scores. Contamination and multicopy genes in the library were filtered first. The RepeatScout library was used for RepeatMasker. We repeated the program to identify homologs in the genome and to categorize the identified repeats.

Moreover, we estimated insertion times for the full-length LTR retrotransposons identified above. We first aligned the 5′- and 3′- LTR sequences of the retrotransposons and used these to calculate the average number of substitutions per aligned site (the *K*-value) using the EMBOSS (6.5.7.0) package distmat. Insertion times (*T*) were calculated according to the following formula: $T\,=\,K/(2\,\times\,r),$, where *r* represents the average substitution rate, in this case 1.3×10^−8^ substitutions per synonymous site per year^47^.

### ChIP cloning

Nuclei were isolated from young leaves according to previously published protocols with minor modification^14^. We purified nuclei twice by sucrose density gradient centrifugation. ChIP experiments using the rabbit antibody against rice CENH3 were performed as described previously^34^. Immunoprecipitated DNA was extracted with phenol/chloroform and precipitated with ethanol. The extracted DNA was converted into blunt-ended DNA fragments by filling in or trimming back 3′ and 5′ overhangs. Next, we phosphorylated 5′ ends and added A-overhangs to 3′ ends using the NEBNext^®^ Ultra™ DNA Library Prep Kit for Illumina (NEB, USA). Modified DNAs were cloned into the pMD19-T vector (TAKARA, Japan), and clones with an insert size larger than 150 bases were sequenced.

### FISH and Immuno-FISH

The preparation of meiotic pachytene chromosomes and subsequent FISH analysis were the same as described previously^14^. Briefly, we labeled probes from clones with inserts larger than 1000 bp with digoxigenin-dUTP. *RIRE2* probes were labeled with Biotin-dUTP. Polyclonal rabbit antibodies against rice CENH3, with a concentration of 1 μg per μL, was used to label centromeric regions on chromosomes. Chromosomes were counterstained with DAPI. Images of hybridization signals and chromosomes were captured using an Olympus BX51 fluorescence microscope system (Olympus, Japan) equipped with a micro CCD camera. Preparation for meiotic pachytene chromosomes and procedures for fluorescence immunolocalization are similar to a previously described protocol with minor modifications^35^. Anthers at pachytene stage were directly fixed in 4% (w/v) paraformaldehyde for 50 min at room temperature. After recording immunostaining signals, slides were washed with PBS buffer and FISH was carried out as described with the indicated probes.

### Centromere sequence screening and annotation

Sequences of centromere-specific probes identified by FISH were used to screen the centromeric regions of *O. granulata*. BLAST searching of the assembled genome of *O. granulata* using sequences of centromere-specific probes was performed under the following conditions: that with the matched lengths matched greater than 1000 bp and similarity higher than 95%. A total of 65 contigs were identified, which resulted in the formation of a 7.36 Mb sequence library representing the centromeric sequences in *O. granulata*. The sequence data were consequently used for centromeric elements analysis. Repeat sequences were identified as follows: Tandem repeats were searched using Tandem Repeats Finder (4.09) (http://tandem.bu.edu/trf/trf.html) and TEs were predicted using RepeatMasker (4.0.6) (http://www.repeatmasker.org/).

### Gene annotation

The *O. granulata* protein-coding gene set was inferred using de novo, homologous and evidence-based gene prediction (i.e., RNA-seq data) approaches. We performed de novo gene prediction on a repeat-masked genome using three programs, namely Augustus (3.0.3)^48^, GlimmerHMM (3.0.1)^49^, and SNAP (version 11/29/2013)^50^. Training models were generated from a subset of the transcriptomic data set representing 800 distinct genes. Homologous gene prediction was performed by comparing protein sequences of *Arabidopsis thaliana* (TAIR10), *Brachypodium distachyon* (v1.0), *O. brachyantha* (v1.4b), *O. glaberrima* (AGI1.1), *Oryza indica* (ASM465v1), *O. sativa* (v7.0), and the uniprot sprot database (release-2015_04). For each reference, we performed the following steps. First, we predicted putative homologous genes from protein sequence alignments representing the complete gene set (the longest transcripts were chosen to represent each gene) with TBLASTN 2.2.18 (*e* value cut-off: 1e-5)^51^. Second, the corresponding regions were retrieved, together with sequences 2 kb downstream and upstream of the aligned regions. Third, we further processed the alignments using GeneWise (2.2.0) to extract accurate exon and intron information^52^. Evidence-based gene prediction was conducted through alignment of all RNA-seq data generated herein against the assembled genome using TopHat (2.0.12)^53^, with cDNAs predicted from the resultant data using Cufflinks (2.2.1)^54^. The resulting cDNA sequences were then aligned to the *O. granulata* genome using BLAT (v. 34 × 12). Following gene prediction, a nonredundant gene set representing homologous genes, de novo genes, and RNA-seq supported genes, was generated using EVidenceModeler (EVM)^55^. In addition, transposase proteins were filtered when a combination of the following conditions is true. First, the protein function was identified as transposase in swissprot or InterPro database (relevant terms include transpose, transposon, Retrotransposon, retrovirus, retrotransposon, reverse transcriptase, transposase, and retroviral). Second, the protein sequences were searched against the transposase database (BLASTP *e* = 1e-10) and sequences with matches were excluded. tRNA genes were searched for using tRNAscan-SE^56^. For rRNA identification, we downloaded *Oryza* rRNA sequences from NCBI and aligned them against the *O. granulata* genome to identify possible rRNAs. Additionally, other types of noncoding RNAs, including miRNA and snRNA, were identified utilizing INFERNAL to search the Rfam database^57^.

### Functional annotation

We performed predicted gene annotation in *O. granulata* by aligning sequences against a number of protein sequence databases including InterPro (54.0), Gene Ontology, KEGG (version 59)^58^, Swiss-Prot (release-2015_04)^59^, TrEMBL (release-2015_04), and NR (07/03/2015). First, to predict protein-coding genes in the *O. granulata* genome, each inferred amino acid sequence was assessed for conserved protein domains in the gene3d, hamap, pfam, pirsf, prints, prodom, smart, superfamily, and tigrfam databases using InterProScan^60^. The Gene Ontology IDs for each gene were obtained from the corresponding InterPro entries. Second, amino acid sequences were subjected to BLASTP (2.2.26) (*e* value < 1e-5) using the following protein databases: Swiss-Prot, TrEMBL, Kyoto Encyclopedia of Genes and Genomes (KEGG), and NCBI protein NR.

### Phylogenetic analysis

Phylogenetic analysis of the seven sequenced *Oryza* genomes was performed using 1:1 single-copy orthologous genes. OrthoMCL clustered a total of 3746 single-copy gene families, which were individually aligned with MUSCLE and then subjected to phylogenetic analyses by MrBbayes with using *A. thaliana* as outgroup^61^. These genes robustly resolved phylogenetic relationships among the five *Oryza* species genomes with all the nodes obtaining full (100%) bootstrap support. Divergence time was conducted with PAML McMcTree^62^, and the following constraints were used for time calibrations: 40–53 million years ago (MYA) for the *Brachypodium*-*Oryza* split^63^ and 15–24 MYA for the origin and divergence of *Oryzeae*^64^. Divergence time dating suggested a recent radiation of about (~ 3.9 MYA) within major AA-genome lineages and a second split (~12.0 MYA) between the GG and FF genomes. Based on the estimated divergence times of the seven species and the inferred phylogenetic tree, the expansion and contraction of gene clusters were determined by a CAFE calculation (version 2.1) on the basis of changes in gene family size in generated phylogenetic history^65^.

### Gene collinearity

Syntenic blocks between *O. granulata* and *O. sativa* were defined by McScan analysis based on core-orthologous gene sets identified using InParanoid (BLAST *e* value ≤ 1e -5; number of genes required to call synteny ≤5)^66^. We confirmed that syntenic blocks represented the orthologous blocks identified between *O. granulata* and *O. sativa*. We then classified genes as either collinear or noncollinear according to whether they had a homologous gene within the orthologous regions. If we failed to detect a homologous gene in the syntenic region of the target genome, we searched for DNA sequences homologous to the candidate gene within this region. We then assigned the syntenic status “without synteny” for this gene if sequence remnants were detected, indicating the orthologous gene was probably mis-annotated and the synteny status of this gene is unclear. To minimize the influence of sequence gaps on synteny analysis, we manually inspected gap-containing and gap-flanking genes to confirm their synteny status and incorporated these results into our synteny analysis. In this case, we used *B. distachyon* as the outgroup to filter candidate noncollinear genes that were found to be collinear with the outgroup.

### Gene family identification

We used a comparative analysis to examine the rate of protein evolution and the conservation of gene repertoires among orthologs in the genomes of *A. thaliana* (TAIR10), *B. distachyon* (v1.0), *O. brachyantha* (v1.4b), *O. glaberrima* (AGI1.1), *O. indica* (ASM465v1), *O. sativa* (v7.0), and *O. granulata*. First, we aligned all-to-all proteins using BLASTP (*e* value of 1e-5). Genes were then clustered using OrthoMCL (1.4) with a Markov inflation index of 1.5 and a maximum *e* value of 1e-5^67^. On this basis, all gene families were ascertained from the seven reference genomes. Furthermore, based on pairwise BLASTP alignment of *O. granulata* and *O. sativa* with *e* value of 1e-5, we used the reciprocal best method to identify orthologous genes, called reciprocal best ortholog gene pairs, which showed high similarity at the amino acid level.

### Enrichment analysis

For a given gene list, such as the *O. granulata*-specific genes or conserved genes between *O. granulata* and *O. sativa*, GO enrichment analysis was used. GOstat tests for GO terms represented by significantly more genes in a given gene set using chi-square test. Fisher’s exact test is used when expected counts are below 5, which makes the chi-square test inaccurate. The computed *p* value was then adjusted for multiple tests by specifying a false discovery rate (FDR) (*q* value < 0.05) using the Benjamini−Hochberg method^68^. Similar methods were also used for IPR and KEGG enrichment analysis.

### Positive selection analysis

We investigated the molecular evolution of protein-coding genes using the set of high confidence 1:1 orthologous genes identified in the seven *Oryza* species. We first compared the average evolutionary rates of protein-coding genes along lineages and clades of the seven species phylogeny. We estimated branch-specific *ω* values (nonsyonymous−synonymous rate ratio, *d*N/*d*S) using the codonML program in PAML (version 4.4)^69^. The two models with both a single *ω* estimated for all branches and a branch-specific *ω* for each branch were compared in a likelihood ratio test (LRT). For all measurements, codon frequencies were estimated from nucleotide frequencies at each codon position (model F3×4). In order to identify genes under positive selection, we performed CodeML and a series of LRTs to calculate the ratio of synonymous (*d*S) and nonsynonymous (*d*N) changes at each codon or on particular branches or clades of interest in the phylogeny for each orthologous family. We corrected for multiple comparisons following the method of Benjamini and Hochberg^68^ to estimate the appropriate *P* value threshold for an FDR of <0.05. Phylogenetic relationships were determined based on the tree constructed above.

### Data availability

The raw sequencing data of RNA-seq and whole-genome have been deposited at NCBI under the BioProject accession numbers PRJNA385906 and PRJNA412686, respectively. The RNA-seq reads of roots, stems, sheaths, panicles, and leaves can be accessed under Sequence Read Archive (SRA) accession numbers SRR5527094 to SRR5527098, respectively. The whole-genome reads and fosmid sequences can be accessed under SRA accession numbers SRR6175404 to SRR6175406. The whole-genome assembly has been depositd in the Genome Warehouse in BIG Data Center under accession number GWHAAEL00000000 (http://bigd.big.ac.cn/gwh).

## Electronic supplementary material

Supplementary Information

Description of additional supplementary items

Supplementary Data 1

Supplementary Data 2

Supplementary Data 3

Supplementary Data 4

Supplementary Data 5

Supplementary Data 6
