# Supplementary material for: De novo genome assembly of Oryza granulata reveals rapid genome expansion and adaptive evolution
Source: Commun Biol. 2018 Jun 29;1:84. doi: 10.1038/s42003-018-0089-4 (PMC6123737; doi:10.1038/s42003-018-0089-4)
Supplement: Supplementary file 1 — Supplementary Information [file 42003_2018_89_MOESM1_ESM.pdf]

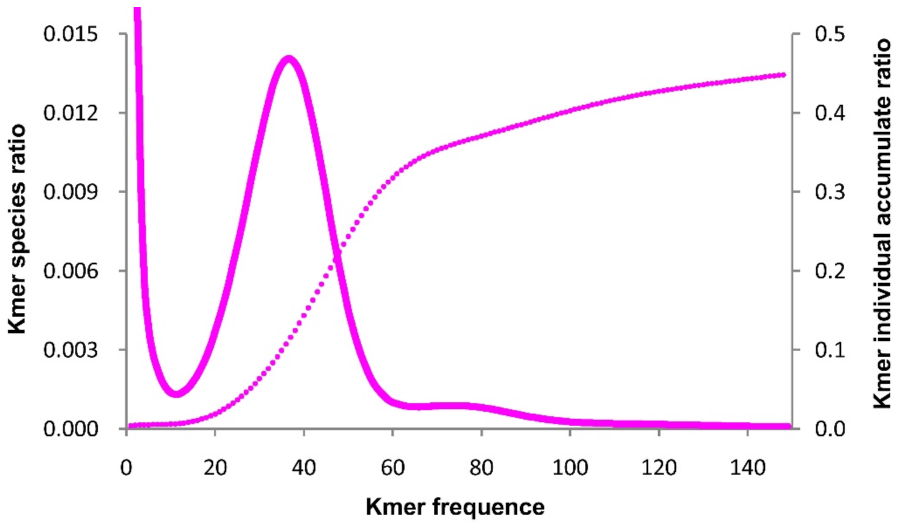

Supplementary Figure 1. *K*-mer (*k* = 17) distribution of *O. granulata*. The solid line represents the ratio of *k*-mer species with difference depths, while the dashed line represents the accumulated ratio of individual *k*-mers.

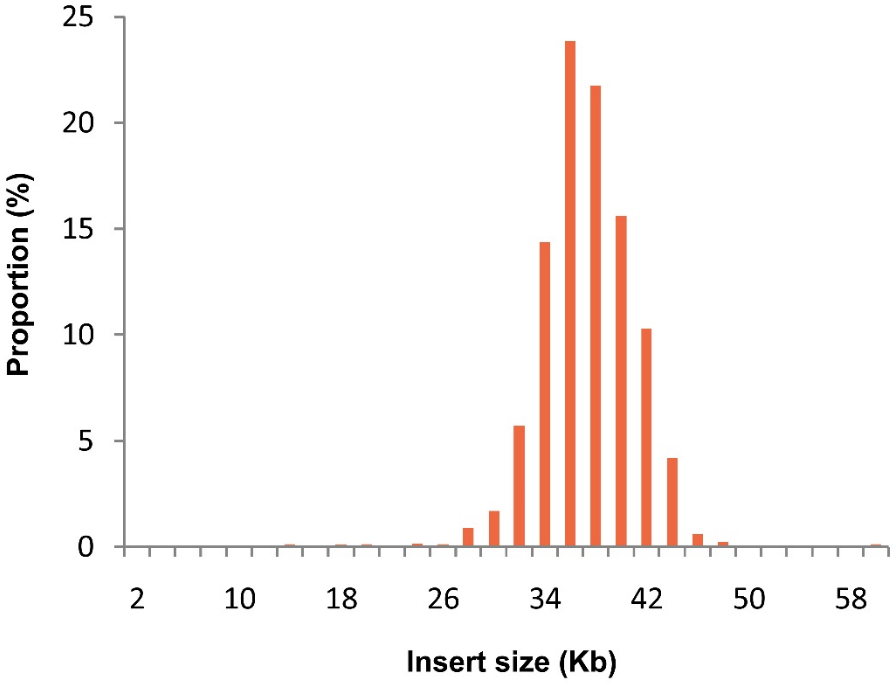

Supplementary Figure 2. Distribution of insert size for mate-pair reads from the fosmid library.

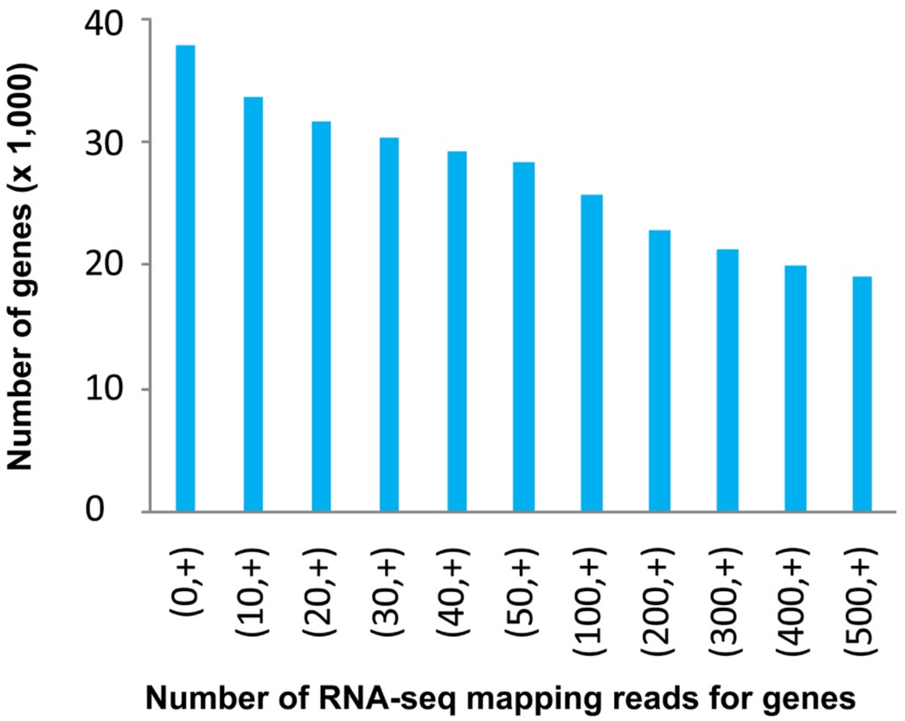

Supplementary Figure 3. Gene coverage using RNA-seq data from five *O. granulata* tissues.

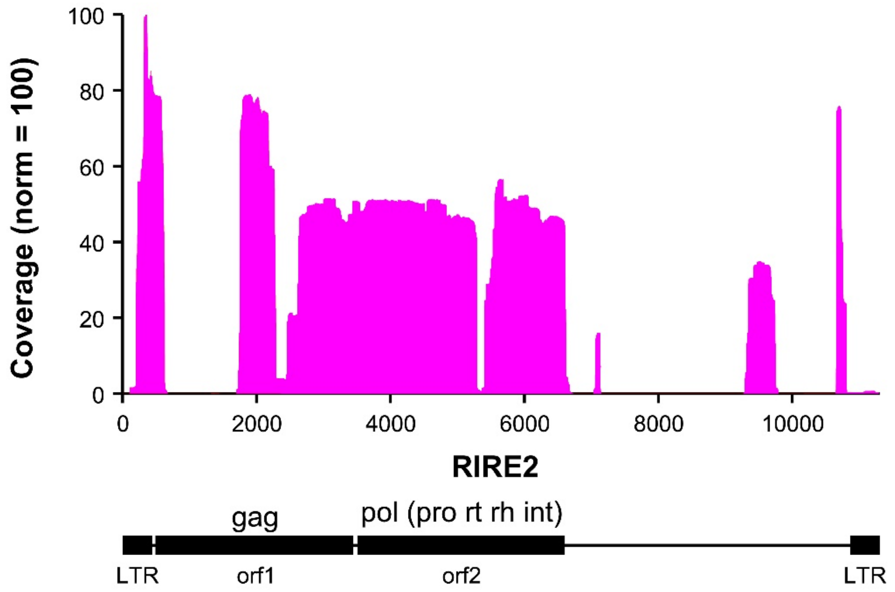

Supplementary Figure 4. Structure and coverage of *RIRE2*. LTR, long terminal repeat; *gag*, the gag gene; *pro*, the protease gene; *rt*, the reverse transcriptase gene; *rh*, the RNase H gene; *int*, the integrase gene.

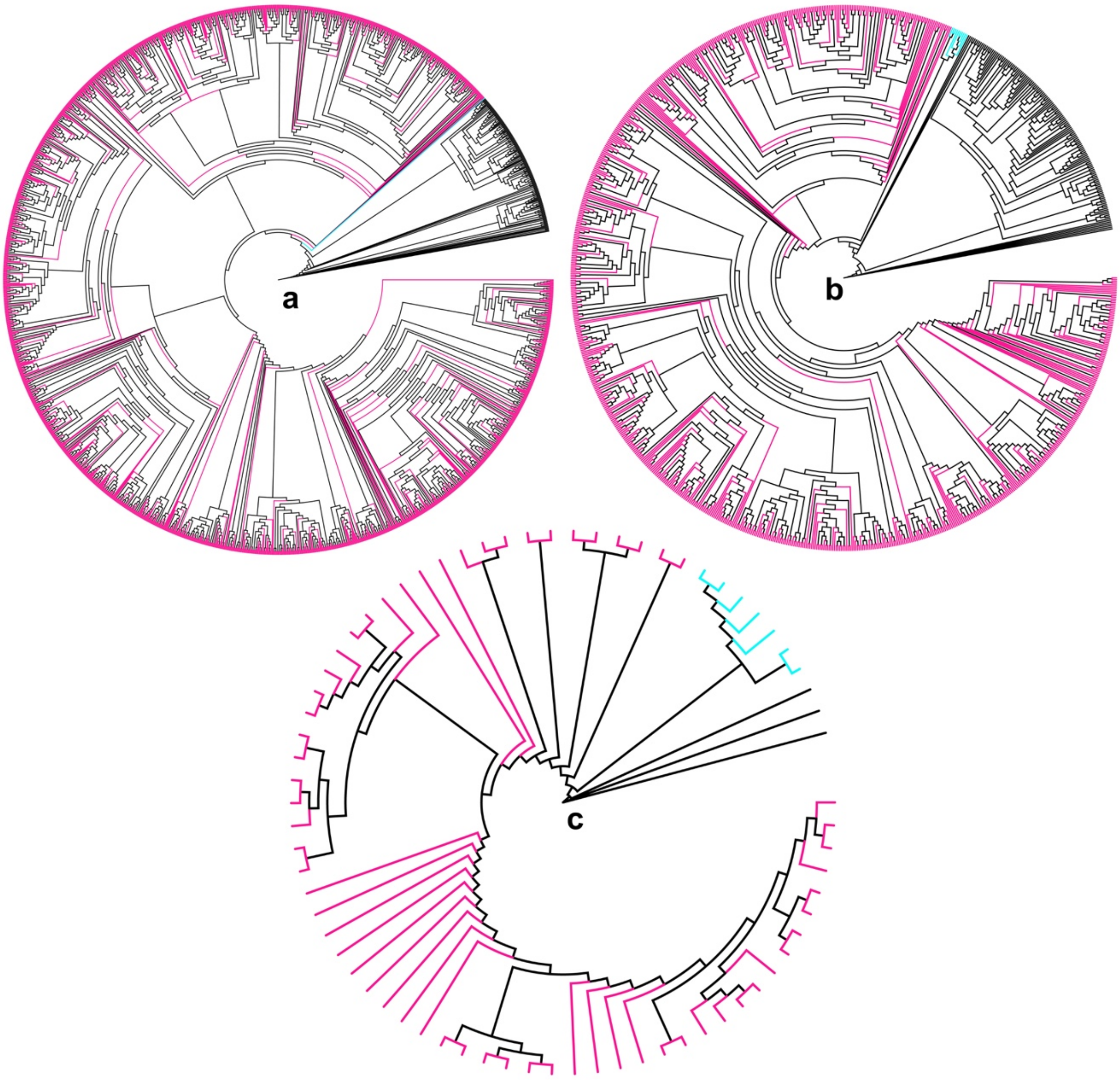

Supplementary Figure 5. Phylogenetic relationships of TE families in three *Oryza* species. Magenta, *O. granulata*; Azury, *O. brachyantha*; and black, *O. sativa.* **a** *RIRE2*_pol TE family; **b** *Atlantys*_OS_p2 TE family; and **c** *Copia*-42_SB_env TE family.

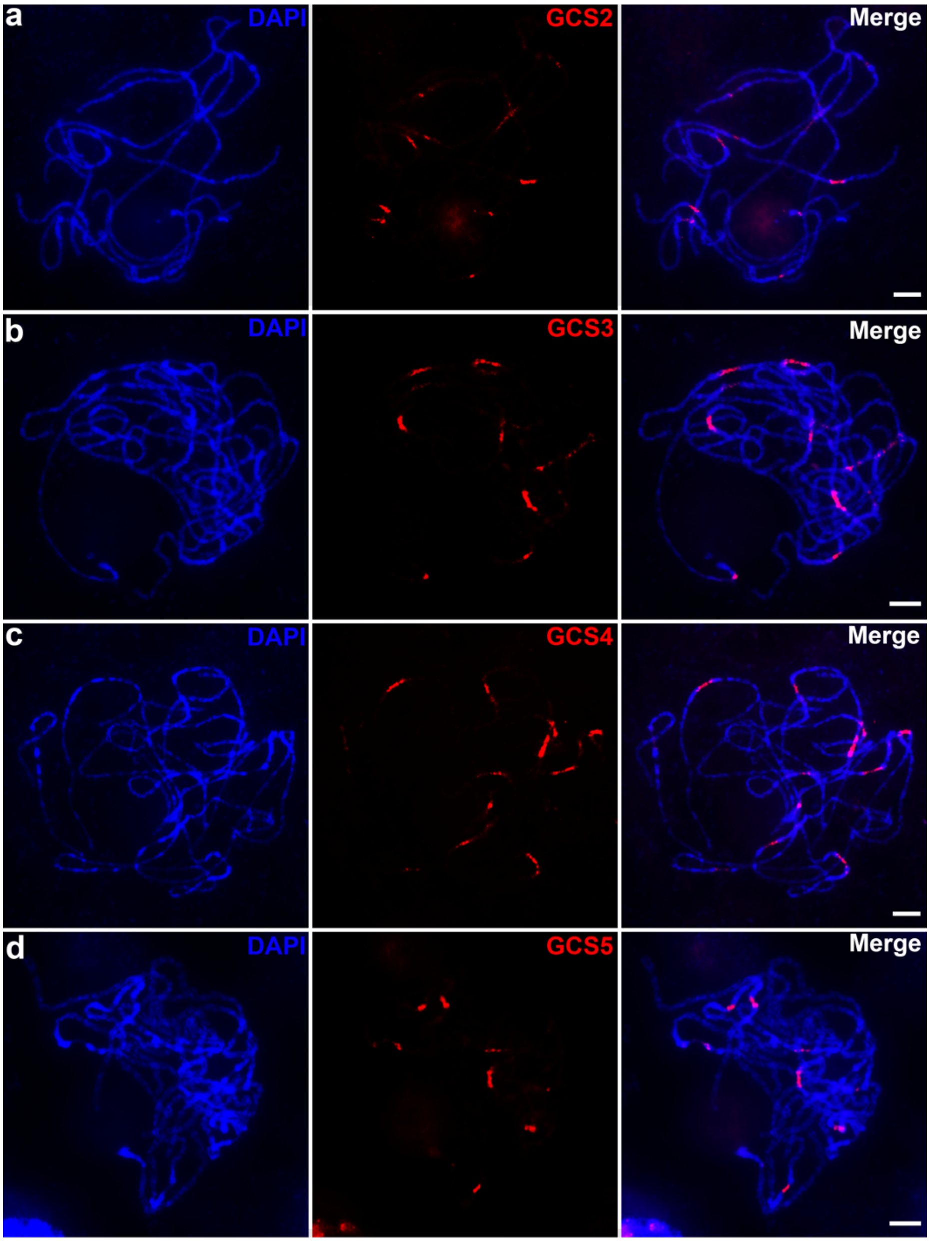

Supplementary Figure 6. FISH identification of centromeric clones from the ChIP-DNA library. **a** GCS2, **b** GCS3, **c** GCS4, and **d** GCS5 were specifically localized in the centromeric regions on pachytene chromosomes of *O. granulata*. Chromosomes were counterstained with DAPI. Probes, GCS2, GCS3, GCS4, and GCS5, were labeled with digoxigenin-dUTP. Scale bars = 5 μm.

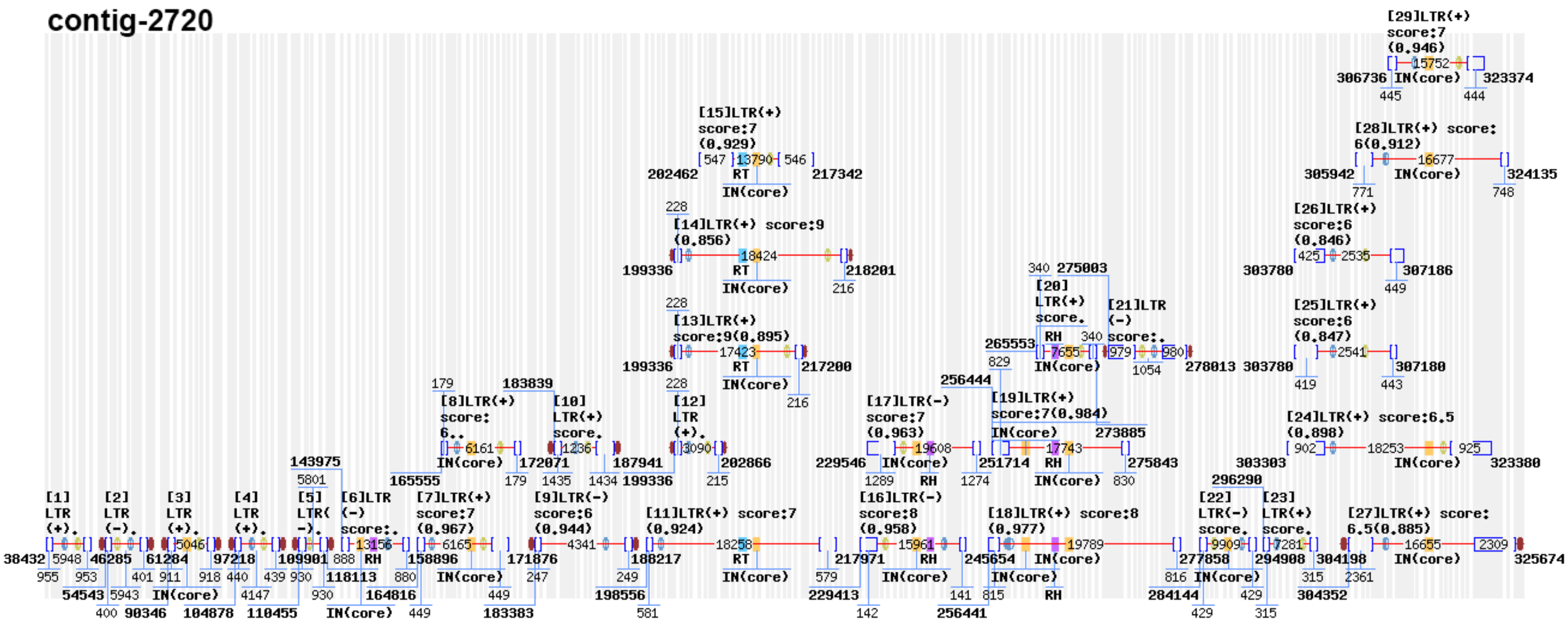

Supplementary Figure 7. Distribution diagram of full-length LTRs within the centromere specific contig 2720. The gray background scale 1:1, 1 base = 1 pixel. The white background scale n: log(n), N bases = c × log(N) pixels; Blue circles denote PBS, brown circles stand for PPT, and purple circles on the end of LTRs represent TSRs.

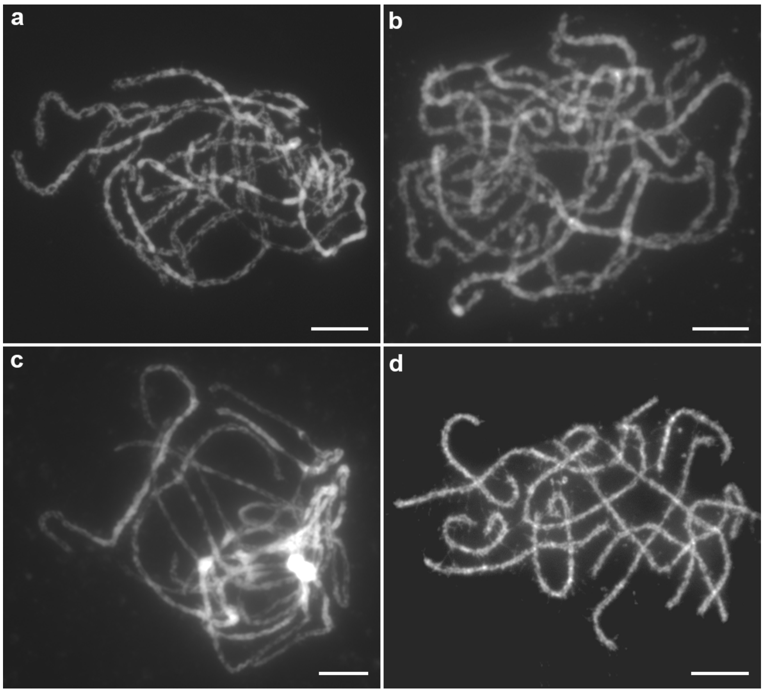

Supplementary Figure 8. Pachytene chromosome morphology of different *Oryza* species. **a** Pachytene chromosomes of *O. punctata* (BB). **b** Pachytene chromosomes of *O. officinalis* (CC). **c** Pachytene chromosomes of *O. australiensis* (EE). **d** Pachytene chromosomes of *O. brachyantha* (FF). Chromosomes were counterstained with DAPI. Scale bars = 5 µm.

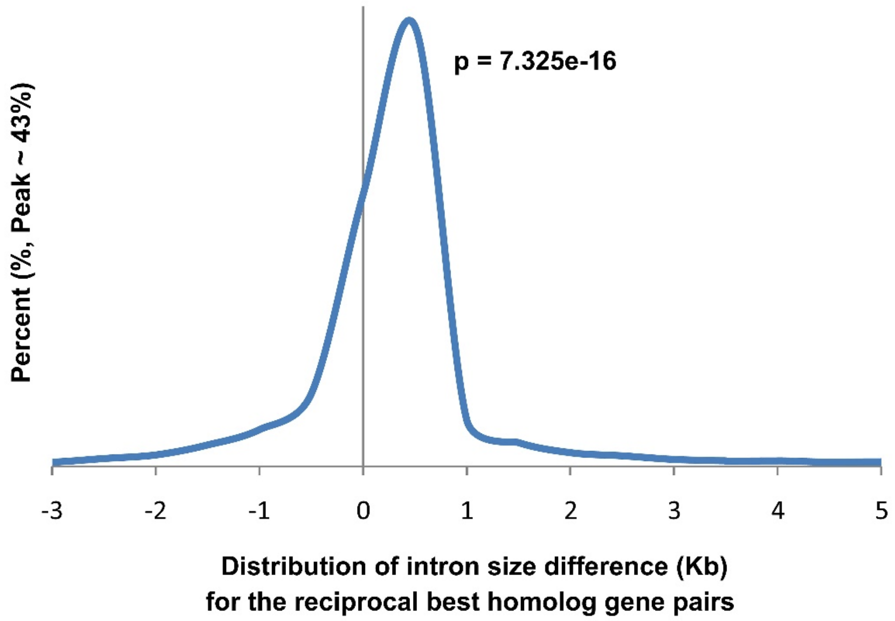

Supplementary Figure 9. Distribution of intron size differences (*O. granulata* - *O. sativa*) with reciprocal best homolog gene pairs between *O. granulata* and *O. sativa*.

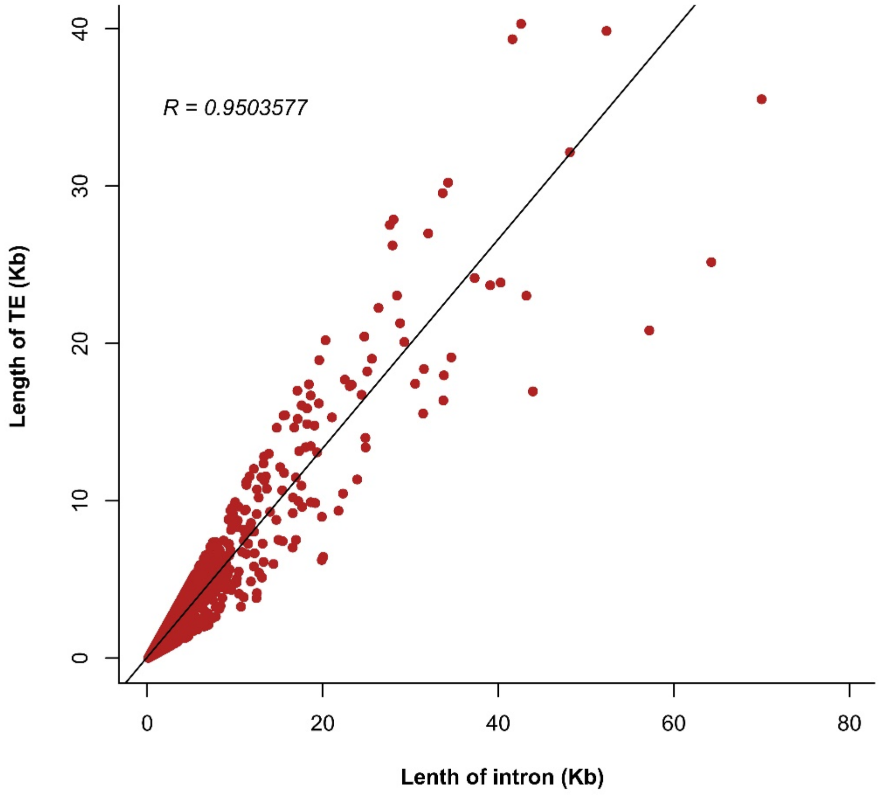

Supplementary Figure 10. Correlation analysis between intron length and intronic TE length in *O. granulata*. The results show that the longer the intron, the stronger the correlation between intron length and intronic TE length, leading to an almost complete correlation in *O. granulata* for the genes with reciprocal best homolog gene pairs between *O. granulata* and *O. sativa*.

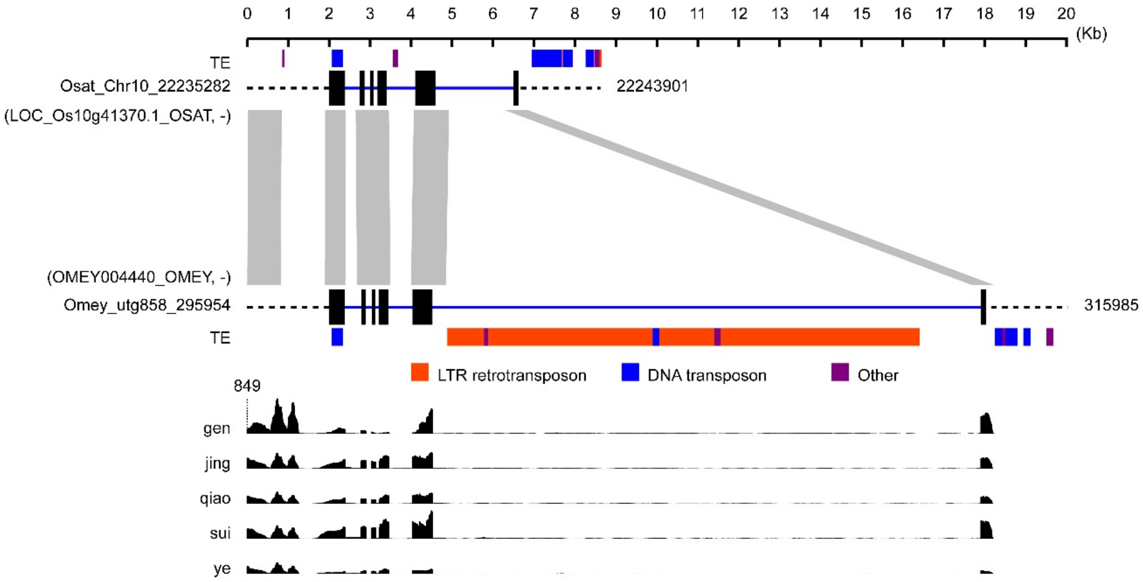

Supplementary Figure 11. Inserts for repetitive sequence with reciprocal best gene pairs (4,620bp vs 16,032bp) between *O. granulata* and *O. sativa*.

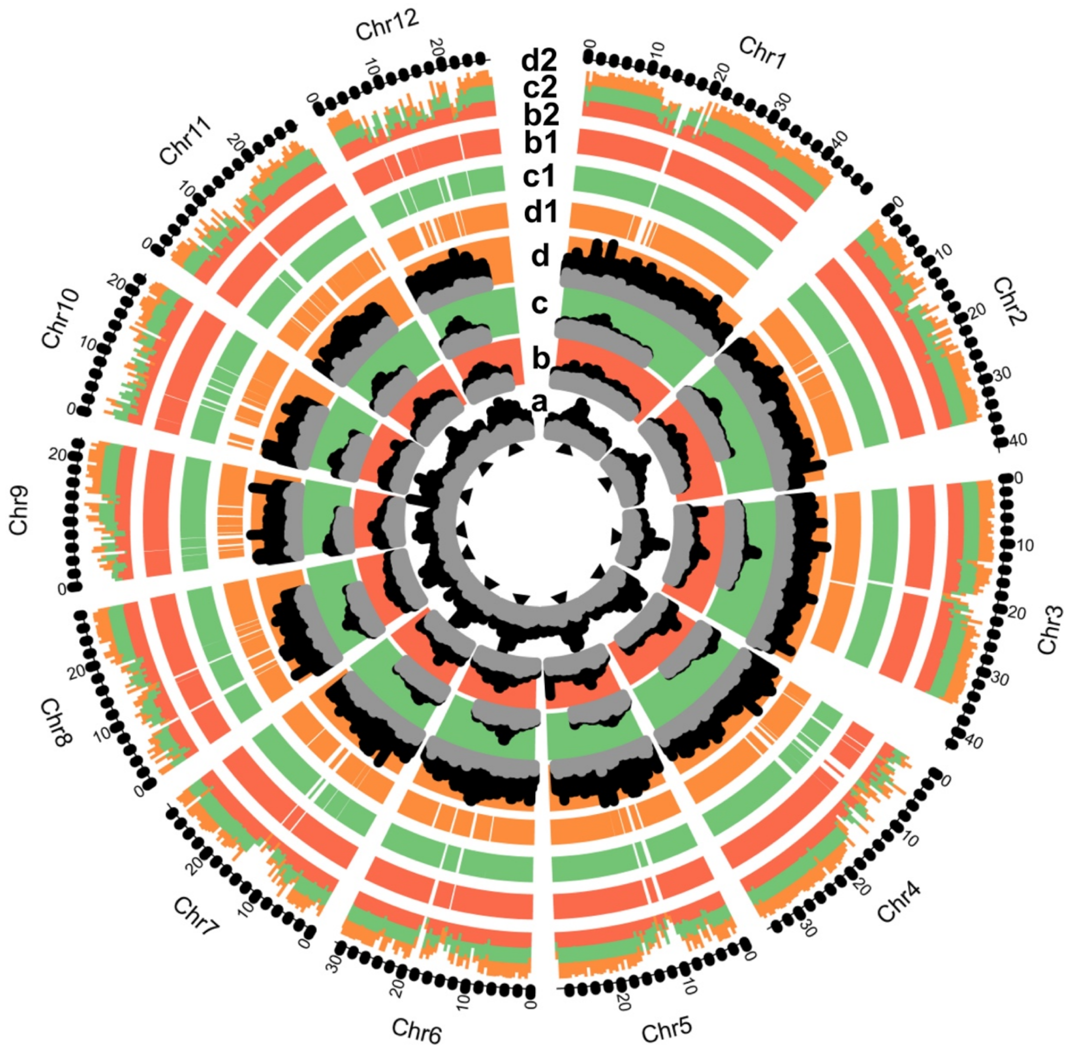

Supplementary Figure 12. Collinear gene blocks between four *Oryza* species. **a**, *O. sativa*; **b**, *O. nivara*; **c**, *O. brachyantha*; and **d**, *O. granulata*. Black and grey represent density of RNA-TE and DNA-TE, respectively. **b1**, **c1**, and **d1** indicate the coverage area of *O. sativa* with collinear gene blocks. **b2**, **c2**, and **d2** show the coverage area (500 Kb in window) of *O. sativa* with collinear gene blocks.

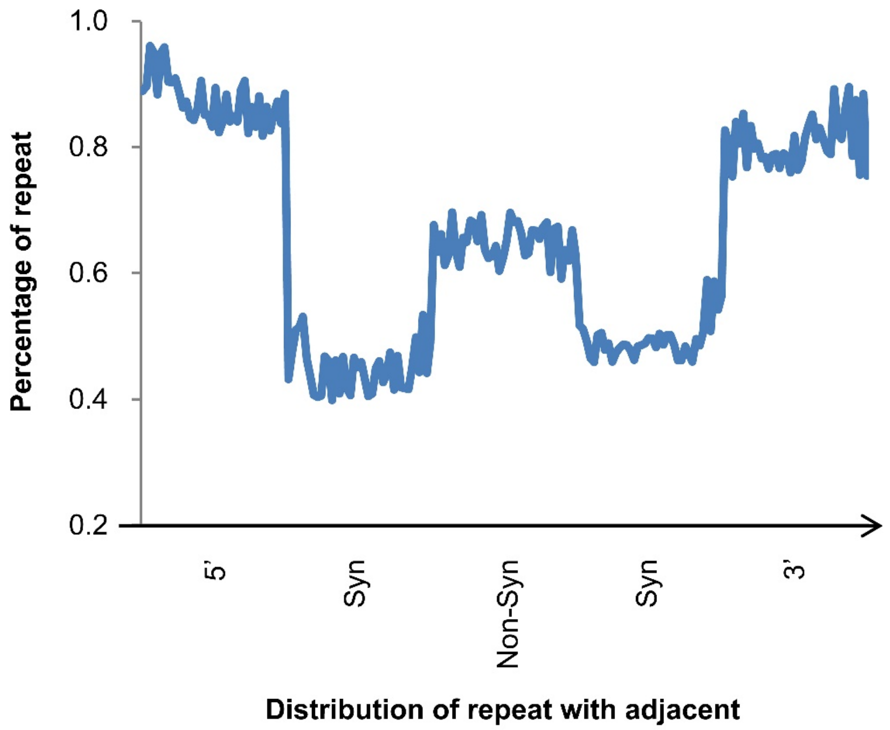

Supplementary Figure 13. Distribution of TEs in adjacent syntenic gene blocks between *O. granulata* and *O. sativa*. Here 40 windows (mean of masked proportion of all the windows) for the five region (5', Syn, Non-Syn, Syn, 3').

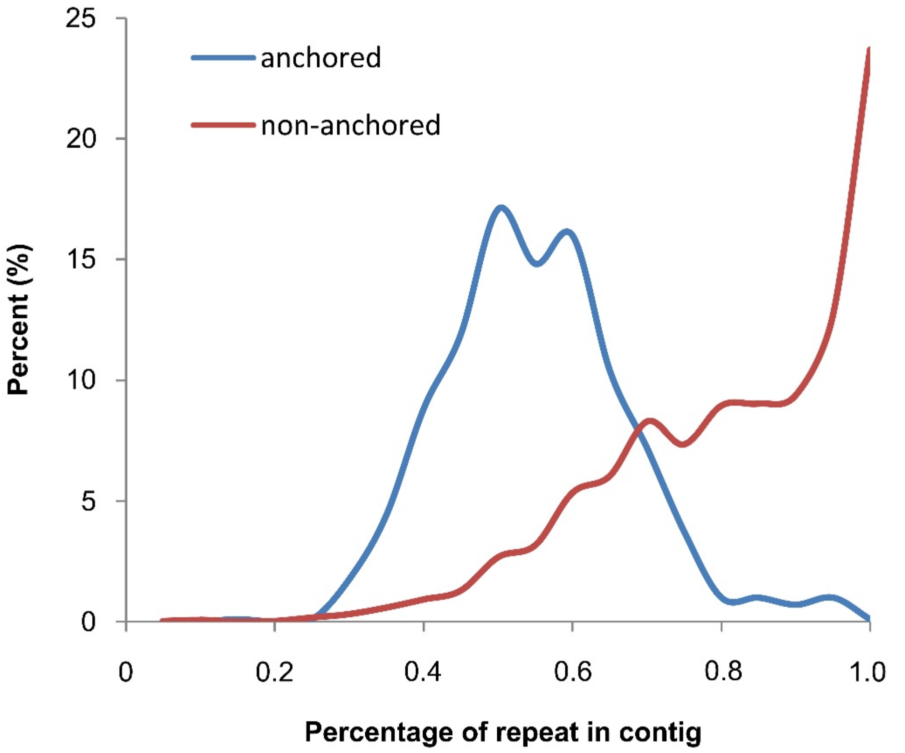

Supplementary Figure 14. Distribution of repeats with anchored and non-anchored contigs in collinear gene blocks of *O. granulata* and *O. sativa*. Repetitive and low complexity sequences were identified and masked using RepeatMasker in 1,019 anchored and 3,599 un-anchored contig assemblies of *O. granulata*. The percentage of the repeat-containing for each contig can be inferred from masked size divided by total size, and the corresponding proportional distribution was calculated.

Supplementary Table 1. Statistics of clean data for *de novo* assembly.

	Sequence Type	Ave Read Length (bp)	Number of Raw reads (Bp)	Number of Raw bases (Bp)	Insert Size	Mapping Ratio (%)
Illumina	pair-end	150	68,593,636	10,289,045,400	~260bp	97.23
		150	68,593,636	10,289,045,400		
		249.1	72,048,850	17,947,939,477	~560bp	99.38
		196.8	72,048,850	14,180,319,573		
		253.5	95,042,633	24,090,906,933	~580bp	99.13
		245.1	95,042,633	23,290,585,986		
	mate-pair	139.4	18,259,592	2,545,665,731	~40Kb	98.9
		138.2	18,259,592	2,523,677,387		
Pacbio	single-end	8,755	1,897,750	16,615,482,433	~20Kb	97.84
Total				121,772,668,320		

Assuming the genome size is 800Mb. Totally, the data is ~121 Gb and ~150X.

Supplementary Table 2. Statistics of the published *Oryza* species.

Species	***O. granulata***	*O. brachyantha*	*O. sativa ssp. japonica*	*O. sativa ssp. indica*	*O. glaberrima*	*O. barthii*	*O. glumaepatula*	*O. meridionalis*	*O. nivara*
Category	**Wild**	Wild	Cultivated	Cultivated	Cultivated	Wild	Wild	Wild	Wild
Genotype	**GG**	FF	AA	AA	AA	AA	AA	AA	AA
Chromosome	**2n = 24**	2n = 24	2n = 24	2n = 24	2n = 24	2n = 24	2n = 24	2n = 24	2n = 24
Overall									
flow cytometry estimated genome size (in Mb) *1	**882**	362	389		357				448
Total scaffolded assembly size (Mb); total scaffolds	**776.96; 4,618**	260.84; 7,485	374.47; 14	427.00; 10,490	316.42; 1,951	308.27; 12	372.86; 12	335.67; 12	337.95; 12
Total scaffolds of > 2kb: length (Mb); no. of scaffolds	**776.96; 4,617**	259.11; 336	374.47; 14	427.00; 10,486	316.41; 1,945	308.27; 12	372.86; 12	335.67; 12	337.95; 12
Total orphan contigs: length (Mb); no. of contigs *2	**776.96; 4,618**	2.27; 7,182	0.00; 0	25.11; 6,302	6.99; 1,204	0.00; 0	0.00; 0	0.00; 0	0.00; 0
Largest scaffold (Mb)	**1.59**	33.92	43.27	47.28	33.05	36.92	46.53	41.80	42.85
Gaps, combined length (kb)	**0.00**	33.92	165.25	16344.67	13104.71	2601.74	71593.11	80041.16	27955.61
N50 in kb of scaffolds; count > N50 length	**262.05; 901**	21479.43; 5	29958.43; 6	31240.96; 6	23192.81; 6	25711.81; 5	31548.19; 5	30391.02; 5	28646.06; 5
N90 in kb of scaffolds; count > N90 length	**75.23; 3,000**	14643.57; 11	23207.29; 11	17.04; 353	16910.67; 12	19274.05; 11	23490.08; 11	18641.91; 11	20407.41; 11
CEG completeness in assembly: complete; partial (%) *3	**95.16; 97.98**	95.97; 97.98	95.16; 97.98	95.56; 98.79	88.31; 93.15	95.97; 97.98	94.76; 98.79	92.74; 95.56	94.35; 97.58
Complete Single-copy BUSCOs (%) *4	**95**	95	96	98	93	96	97	86	97
Protein-coding regions									
Number of gene models *5	**40,116**	32,023	42,849	40,733	31,181	34,501	35,657	29,248	36,245
Gene density (genes per Mb)	**51**	122	114	95	98	111	95	87	107
Exonic proportion, including introns (bp)	**102,744,739**	81,735,004	95,539,047	99,378,473	74,099,226	104,629,468	123,534,711	94,684,805	116,397,900
Mean/Median gene size (bp)	**2561.19/1486**	2552.38/1760	2229.67/1557	2439.75/1694	2376.42/1679	3032.65/2050	3464.53/2167	3237.31/2106	3211.42/2128
Mean/Median CDS size (bp)	**1012.28/783**	1061.51/816	1023.10/795	1117.30/912	1088.03/897	1134.00/840	1145.82/882	1168.00/894	1160.72/888
Number of exons	**162,369**	146,744	169,448	170,465	133,591	168,298	169,053	141,505	171,429
Number of bp included in exons	**40,608,470**	33,992,712	43,838,894	45,510,996	33,926,006	39,124,214	40,856,639	34,161,672	42,070,116
Mean/Median exon size (bp)	**250.10/146**	231.65/132	258.72/141	266.98/144	253.95/138	232.47/133	241.68/132	241.42/134	245.41/134
Mean number of exons per gene	**4.05**	4.58	3.95	4.18	4.28	4.88	4.74	4.84	4.73
Number of introns	**122,253**	114,721	126,599	129,732	102,410	133,797	133,396	112,257	135,184
Number of bp included in introns	**62,136,267**	47,742,293	51,700,152	53,867,477	40,173,219	65,505,253	82,678,070	60,523,133	74,327,784
Mean/Median intron size (bp)	**508.26/148**	416.16/162	408.38/173	415.22/171	392.28/154	489.59/178	619.79/188	539.15/182	549.83/187
Overall G + C content (%)	**46.32**	40.50	43.57	43.48	42.85	42.10	42.43	42.83	42.40
Exons, G + C content (%)	**52.17**	53.20	53.15	55.20	54.75	53.80	55.12	55.26	55.12
Introns, G + C content (%)	**42.01**	37.98	38.83	37.60	37.07	37.79	38.48	38.17	38.08
Intergenic regions, G + C content (%)	**46.34**	39.50	41.67	42.88	42.33	41.22	41.62	42.07	41.44

*1, From Ammiraju, J. S. et al. (2006)

*2, Orphan contigs indicates not any gaps in the scaffolds.

*3, A survey of the core eukaryotic genome (CEGs) was conducted to assess the completeness of the protein coding portions of the assemblies.

*4, Assessing genome assembly completeness with Benchmarking Universal Single-Copy Orthologs.

*5, For protein-coding, need to satisfy the conditions: aa > 30bp; non-pseudogene; keep only the longest transcript for one gene.

Supplementary Table 3. Estimation of *O. granulata* genome size based on 17 *K*-mer statistics.

K-mer	*K*-mer Number	Peak Depth	Genome Size
17	29,049,891,363	37	785,132,199

Supplementary Table 4. Evaluation of the *O. granulata* genome completeness using clean data set of RNA-seq.

Tissue	Number of Raw reads	Number of Raw bases	PE Mapping Ratio (%)
root	40,905,511	5,815,232,376	92.7
	40,905,511	5,735,238,651	
stem	46,204,743	6,592,362,362	93.22
	46,204,743	6,496,212,188	
sheathe	42,739,186	6,090,441,401	93.46
	42,739,186	6,010,203,640	
panicle	39,472,253	5,623,174,721	92.32
	39,472,253	5,553,001,415	
leaf	42,788,780	6,089,181,166	92.77
	42,788,780	6,043,231,054	

Supplementary Table 5. Summary of functional annotation of predict genes in *O. granulata*.

	Number	Percent (%)
Total	40,116	
InterPro	28,621	71.35
GO	19,999	49.85
KEGG	18,825	46.93
Swissprot	23,696	59.07
TrEMBL	33,803	84.26
Annotated	33,901	84.51
Unanotated	6,215	15.49

Supplementary Table 6. Summary of non-coding RNA annotation of *O. granulata*.

Type	Copy Number	Average length(bp)	Total length(bp)	% of genome
miRNA	233	132.13	30,787	0.00
tRNA	1,083	75.79	82,079	0.01
rRNA	722	137.53	99,297	0.01
18S	54	653.24	35,275	0.00
28S	80	123.24	9,859	0.00
5.8S	20	139.05	2,781	0.00
5S	568	90.46	51,382	0.01
snRNA	512	115.40	59,084	0.01
CD-box	399	108.12	43,138	0.01
HACA-box	53	128.11	6,790	0.00
splicing	60	152.60	9,156	0.00

Supplementary Table 7. Repeats in Genome of *O. granulata*, *O. sativa* and *O. brachyantha*.

	O. granulata		*O. sativa*		*O. brachyantha*	
Genome Size (bp)	776,957,504		373,245,519		260,838,168	
Type	Repeat Size	% of genome	Repeat Size	% of genome	Repeat Size	% of genome
Trf	18,791,063	2.42	12,979,041	3.48	4,956,759	1.90
Repeatmasker	295,468,491	38.03	139,830,367	37.46	44,339,064	17.00
Proteinmask	200,868,027	25.85	43,853,094	11.75	11,172,146	4.28
De novo	474,953,618	61.13	140,176,567	37.56	78,478,483	30.09
Total	528,042,583	67.96	169,918,394	45.52	85,923,344	32.94

Supplementary Table 8. Common and specific high-frequent (depth >=150) *k*-mer in *O. granulata* and *O. sativa* genome.

Kmer Size	23		33		43		53		63	
Species	*O. sativa*	*O. granulata*	*O. sativa*	*O. granulata*	*O. sativa*	*O. granulata*	*O. sativa*	*O. granulata*	*O. sativa*	*O. granulata*
Same Kmer Num	2,156,818,832	2,438,304,387	1,654,564,615	1,878,130,905	1,319,643,632	1,571,660,469	1,053,653,721	1,345,993,555	831,169,272	1,160,321,861
Same Kmer aveDepth	5991.9	6773.9	6667.68	7568.62	6233.94	7424.45	5570.76	7116.39	4832.77	6746.61
Same Kmer Num(%)	38.42	12.19	37.74	12.68	37.67	13.92	37.46	15.17	37.06	16.19
Same Kmer Species	359,956	359,956	248,147	248,147	211,687	211,687	189,140	189,140	171,986	171,986
Same Kmer Species(%)	8.26	1.79	7.4	1.5	7.98	1.6	8.9	1.8	10.07	2.05
Different Kmer Num	3,456,819,397	17,563,225,396	2,729,381,367	12,933,890,916	2,183,770,401	9,722,038,047	1,759,177,845	7,529,517,449	1,411,512,297	6,005,590,859
Different Kmer aveDepth	864.58	889.86	879.58	791.25	894.27	746.38	908.37	730.07	918.57	730.7
Different Kmer Num(%)	61.58	87.81	62.26	87.32	62.33	86.08	62.54	84.83	62.94	83.81
Different Kmer Species	3,998,248	19,737,115	3,103,038	16,346,211	2,441,950	13,025,611	1,936,637	10,313,487	1,536,633	8,218,960
Different Kmer Species(%)	91.74	98.21	92.6	98.5	92.02	98.4	91.1	98.2	89.93	97.95

Supplementary Table 9. Repeat in Genome of *O. granulata*, *O. sativa* and *O. brachyantha* with different type.

		Repbase TEs		TE proteins		*De novo*		Combined TEs	
		Length (bp)	% in genome	Length (bp)	% in genome	Length (bp)	% in genome	Length (bp)	% in genome
O. granulata	DNA	47,531,100	6.12	24,964,784	3.21	36,430,508	4.69	68,393,246	8.80
	LINE	4,114,272	0.53	4,924,352	0.63	2,263,453	0.29	7,169,231	0.92
	SINE	45,420	0.01	0	0.00	14,853	0.00	59,741	0.01
	LTR	244,297,235	31.44	171,596,609	22.09	437,800,105	56.35	460,976,797	59.33
	Other	6,998	0.00	0	0.00	0	0.00	6,998	0.00
	Unknown	0	0.00	3,339	0.00	1,692,874	0.22	1,696,213	0.22
	Total	295,468,491	38.03	200,868,027	25.85	474,933,150	61.13	522,858,044	67.30
O. sativa	DNA	54,801,858	14.68	9,880,705	2.65	37,858,863	10.14	66,625,386	17.85
	LINE	3,910,057	1.05	4,383,994	1.17	4,801,456	1.29	8,518,809	2.28
	SINE	761,782	0.20	0	0.00	654,496	0.18	1,197,438	0.32
	LTR	80,771,154	21.64	29,679,315	7.95	98,566,040	26.41	104,374,478	27.96
	Other	55,997	0.02	0	0.00	1,111	0.00	57,108	0.02
	Unknown	0	0.00	16,191	0.00	2,113,040	0.57	2,129,231	0.57
	Total	139,830,367	37.46	43,853,094	11.75	139,719,773	37.43	165,790,586	44.42
O. brachyantha	DNA	28,520,485	10.93	2,547,788	0.98	43,382,629	16.63	48,430,813	18.57
	LINE	1,192,597	0.46	1,297,544	0.50	3,011,087	1.15	3,918,286	1.50
	SINE	111,927	0.04	0	0.00	316,297	0.12	397,359	0.15
	LTR	14,756,746	5.66	7,346,911	2.82	28,122,718	10.78	29,715,959	11.39
	Other	7,824	0.00	0	0.00	0	0.00	7,824	0.00
	Unknown	0	0.00	5,321	0.00	6,744,181	2.59	6,749,502	2.59
	Total	44,339,064	17.00	11,172,146	4.28	78,131,721	29.95	83,347,690	31.95

Supplementary Table 10. Comparison of all TE super-types among the genome of *O. granulata*, *O. sativa* and *O. brachyantha*.

supertype	*O. granulata*	Percent	*O. sativa*	Percent	*O. brachyantha*	Percent	*P*-value (*O. granulata* vs *O. sativa*)
LTR/Gypsy	407,036,517	0.523885	83,476,174	0.22365	19,375,344	0.0742811	2.20E-16
LTR/Copia	54,901,814	0.0706626	20,095,858	0.0538409	10,739,635	0.0411736	2.20E-16
LTR/LTR	28,158,459	0.036242	10,231,035	0.027411	788,382	0.00302249	2.20E-16
DNA/MULE	24,670,124	0.0317522	15,033,034	0.0402765	7,889,819	0.0302479	2.20E-16
DNA/MuLE	20,665,533	0.026598	3,267,320	0.00875381	1,328,571	0.00509347	2.20E-16
DNA/CMC	11,837,915	0.0152362	20,985,953	0.0562256	7,055,612	0.0270498	2.20E-16
DNA/hAT	11,760,676	0.0151368	4,808,097	0.0128819	4,029,605	0.0154487	2.20E-16
DNA/PIF	7,781,888	0.0100158	13,635,342	0.0365318	16,709,736	0.0640617	2.20E-16
LINE/L1	6,057,991	0.00779707	8,206,749	0.0219875	3,541,244	0.0135764	2.20E-16
DNA/TcMar	4,185,458	0.00538698	10,566,589	0.02831	9,337,197	0.0357969	2.20E-16
DNA/Helitron	3,283,035	0.0042255	4,555,302	0.0122046	657,427	0.00252044	2.20E-16
DNA/DNA	2,184,724	0.0028119	4,066,211	0.0108942	8,296,248	0.0318061	2.20E-16
LTR/Caulimovirus	2,095,939	0.00269762	599,616	0.00160649	10,620	4.07E-05	2.20E-16
Unknown/Unknown	1,696,213	0.00218315	2,129,231	0.00570464	6,749,502	0.0258762	2.20E-16
LTR/ERV1	1,354,496	0.00174333	185,834	0.000497887	56,048	0.000214877	2.20E-16
LTR/Caulimoviru	1,330,246	0.00171212	397,425	0.00106478	6,388	2.45E-05	2.20E-16

Note: here only showed super-types with total size > 1Mb for any one species.

Supplementary Table 11. Comparison of all TE families among the genome of *O. granulata*, *O. sativa* and *O. brachyantha*.

family	*O. granulata*	Percent	*O. sativa*	Percent	*O. brachyantha*	Percent	*P*-value (*O. granulata* vs *O. sativa*)
LTR/Gypsy/lcl|RIRE2_I-int	57,870,650	0.07448370	3,366,201	0.00901873	603,249	0.00231273	2.20E-16
LTR/Gypsy/RIRE2_gag	33,753,369	0.04344300	979,576	0.00262448	347,900	0.00133378	2.20E-16
LTR/Gypsy/RIRE2_pol	33,292,454	0.04284980	1,014,410	0.00271781	334,105	0.00128089	2.20E-16
LTR/Gypsy/lcl|ATLANTYS-I_OS	18,408,631	0.02369320	2,997,300	0.00803037	670,217	0.00256947	2.20E-16
DNA/MULE-MuDR/lcl|MUDR1_OS	12,844,559	0.01653190	1,325,945	0.00355247	91,699	0.00035156	2.20E-16
LTR/Gypsy/rnd-6_family-505	9,583,560	0.01233470	0	0.00000000	0	0.00000000	2.20E-16
LTR/Gypsy/Atlantys_OS_pol	9,325,595	0.01200270	910,648	0.00243981	397,986	0.00152580	2.20E-16
LTR/Gypsy/Atlantys_OS_gag	8,691,641	0.01118680	928,656	0.00248806	399,758	0.00153259	2.20E-16
LTR/Gypsy/lcl|TRUNCATOR2_OS	8,497,167	0.01093650	2,577,380	0.00690532	45,311	0.00017371	2.20E-16
LTR/Gypsy/RIRE2_p2	8,270,363	0.01064460	312,363	0.00083688	97,524	0.00037389	2.20E-16
LTR/Gypsy/lcl|rn_364-201_IR	7,710,068	0.00992341	142,097	0.00038071	183,133	0.00070209	2.20E-16
LTR/Gypsy/lcl|RETROSAT2LTRA	7,700,783	0.00991146	1,493,559	0.00400155	389,662	0.00149388	2.20E-16
LTR/Gypsy/RETROSOR1_SB	7,046,170	0.00906893	775,433	0.00207754	60,653	0.00023253	2.20E-16
LTR/Gypsy/Gypsy2-HV	7,010,175	0.00902260	667,777	0.00178911	63,262	0.00024253	2.20E-16
LTR/Gypsy/lcl|SZ-48_LTR	6,320,830	0.00813536	938,893	0.00251548	17,112	0.00006560	2.20E-16
LTR/Gypsy/rnd-3_family-13	5,995,617	0.00771679	0	0.00000000	0	0.00000000	2.20E-16
LTR/Gypsy/lcl|SZ-44_LTR	5,927,852	0.00762957	626,303	0.00167799	137,129	0.00052572	2.20E-16
LTR/Gypsy/RETROSAT2_gagpo	5,286,676	0.00680433	1,123,820	0.00301094	596,646	0.00228742	2.20E-16

Note: here only showed super-types with total size > 5Mb for any one species.

Supplementary Table 12. Example for comparison of TE families among the genome of *O. granulata*, *O. sativa* and *O. brachyantha*.

Family	*O. granulata*	Percent	*O. sativa*	Percent	*O. brachyantha*	Percent	*P*-value (*O. granulata* vs *O. sativa*)
LTR/Gypsy/lcl|RIRE2_I-int	57,870,650	0.07448370	3,366,201	0.00901873	603,249	0.00231273	2.20E-16
LTR/Gypsy/RIRE2_gag	33,753,369	0.04344300	979,576	0.00262448	347,900	0.00133378	2.20E-16
LTR/Gypsy/RIRE2_pol	33,292,454	0.04284980	1,014,410	0.00271781	334,105	0.00128089	2.20E-16
LTR/Gypsy/RIRE2_p2	8,270,363	0.01064460	312,363	0.00083688	97,524	0.00037389	2.20E-16
LTR/Gypsy/lcl|RIRE2_LTR	1,226	0.00000158	304,151	0.00081488	1,837	0.00000704	2.20E-16
	133,188,062	0.17142268	5,976,701	0.01601279	1,384,615	0.00530833	
LTR/Gypsy/lcl|ATLANTYS-I_OS	18,408,631	0.02369320	2,997,300	0.00803037	670,217	0.00256947	2.20E-16
LTR/Gypsy/Atlantys_OS_pol	9,325,595	0.01200270	910,648	0.00243981	397,986	0.00152580	2.20E-16
LTR/Gypsy/Atlantys_OS_gag	8,691,641	0.01118680	928,656	0.00248806	399,758	0.00153259	2.20E-16
LTR/Gypsy/Atlantys_OS_p2	3,214,960	0.00413788	454,799	0.00121850	159,317	0.00061079	2.20E-16
LTR/Gypsy/lcl|ATLANTYS-LTR_OS	155,187	0.00019974	1,677,574	0.00449456	7,932	0.00003041	2.20E-16
	39,796,014	0.05122032	6,968,977	0.01867130	1,635,210	0.00626906	
LTR/Copia/lcl|COPIA2-I_OS	1,725,706	0.00222111	1,012,663	0.00271313	195,859	0.00075088	2.20E-16
LTR/Copia/Copia2_OS	1,381,694	0.00177834	869,447	0.00232942	152,472	0.00058455	2.20E-16
LTR/Copia/lcl|COPIA2-LTR_OS	846,185	0.00108910	611,538	0.00163843	221,659	0.00084980	2.20E-16
LTR/Copia/lcl|OSCopia2_I-int	559,196	0.00071973	126,776	0.00033966	40,445	0.00015506	2.20E-16
LTR/Copia/OSCopia2	527,835	0.00067936	134,582	0.00036057	42,065	0.00016127	2.20E-16
LTR/Copia/lcl|OSCopia2_LTR	7,910	0.00001018	2,234	0.00000599	710	0.00000272	1.14E-06
	5,048,526	0.00649782	2,757,240	0.00738720	653,210	0.00250427	

Supplementary Table 13. Centromere-specific contigs screened out from assembled *O. granulata* genome.

Name	Length (bp)	Name	Length (bp)
contig 15	225762	contig 4802	54877
contig 233	135760	contig 5171	50931
contig 401	165737	contig 5296	36481
contig 761	199648	contig 5495	101970
contig 796	72529	contig 5739	133642
contig 863	30900	contig 5808	76347
contig 1019	90991	contig 6638	136600
contig 1116	58708	contig 8068	76861
contig 1148	158847	contig 8339	285117
contig 1221	42909	contig 10580	68305
contig 1234	73103	contig 10608	106908
contig 1673	91770	contig 10615	52178
contig 1818	31823	contig 11385	84061
contig 1913	96817	contig 11640	42617
contig 1993	22417	contig 12053	40133
contig 2070	487789	contig 12322	59916
contig 2202	46345	contig 12358	43832
contig 2247	130197	contig 12896	105613
contig 2448	129894	contig 13233	48339
contig 2538	211011	contig 13417	74309
contig 2551	161108	contig 13442	38861
contig 2720	337098	contig 13801	104151
contig 2783	36861	contig 15830	78714
contig 3115	194161	contig 19178	42073
contig 3120	21279	contig 20858	16070
contig 3185	231117	contig 21406	27404
contig 3375	242045	contig 22427	47455
contig 3380	100015	contig 23129	51854
contig 3419	79962	contig 23770	38127
contig 3519	68247	contig 26641	38124
contig 3651	83213	contig 28128	43174
contig 3916	444164	contig 32250	43899
contig 4129	215675	contig 5495	101970
contig 4802	54877	contig 5739	133642
contig 5171	50931	contig 5808	76347
contig 5296	36481	Total	7360109
*Blast searching conditions: match length ≥ 1000bp, similarity ≥ 95%.			

Supplementary Table 14. Repeats analysis of the centromeric sequence library of *O. granulata*.

Library size (bp)		7,360,109
Type	Repeat Size (bp)	% of library
Gypsy/DIRS1	2,712,960	36.86
Ty1/Copia	346,903	4.71
DNA transposons	200,958	2.73
LINEs	6,477	0.09
L1/CIN4	6,263	0.09
SINEs	1,452	0.02
Unclassified	21,785	0.30
Total interspersed repeats	3,313,257	45.02
Satellite repeats	600	0.01

Supplementary Table 15. The main LTRs of the centromeric sequence library of *O. granulata*.

Centromere-specific LTRs	Copy number
CRM-LTR_OS	743
Gypsy-10C_OS-LTR	601
CRM-I_OS	323
SZ-22_LTR	275
RIRE2_I-int	274
RIRE7_LTR	186
ATLANTYS-I_OS	185
Gypsy-21_OS-LTR	150
SZ-22_int-int	147
RIREXE_I-int	132
RIRE7_I-int	115
SZ-10_I-int	115
Gypsy-14_OS-LTR	105

*Copy number >100 are listed here.

Supplementary Table 16. Statistics of the reciprocal best homolog genes between *O. granulata* and *O. sativa*.

Species	Reciprocal_best_gene_number	mRNA_length	cds_length	Exon_number	Exon_length	Intron_length	Mean_identity
O. sativa	19,564	3064.39	1381.28	5.44	253.8	378.87	70.41
O. granulata		3268.97	1368.68	5.57	245.78	415.94	

Supplementary Table 17. Comparison of intronic TE families of the reciprocal best homolog genes between *O. granulata* and *O. sativa*.

Family	*O. granulata*	*O. sativa*	Different
LTR/Gypsy/lcl|RIRE2_I-int	142,496	0	142,496
LINE/RTE-BovB/RTE-1_SBi_pol	126,720	537	126,183
DNA/MULE-MuDR/lcl|MUDR1_OS	124,050	517	123,533
LINE/RTE-BovB/rnd-4_family-418	123,303	0	123,303
LTR/Gypsy/RLG_scReina_1_1	111,871	1,089	110,782
LTR/Gypsy/RIRE2_gag	97,670	519	97,151
LINE/RTE-BovB/lcl|RTE-1_TD	87,513	997	86,516
LTR/Gypsy/lcl|SZ-48_LTR	97,414	16,231	81,183
LTR/Copia/lcl|Copia2-SB_I-int	78,684	5,235	73,449
LTR/Gypsy/RIRE2_pol	73,555	819	72,736
LTR/Gypsy/SZ-54A	65,742	528	65,214
DNA/PIF-Harbinger/lcl|TOURIST-XV	86,383	24,693	61,690
LTR/Gypsy/SZ-54	59,633	879	58,754
LINE/RTE-BovB/lcl|RTE1_ZM	58,320	233	58,087
LTR/Copia/Copia-11_BD	70,641	13,992	56,649
LINE/RTE-BovB/RTE-1_Mad_pol	53,251	0	53,251

Note: here only listed families with different size > 50Kb.

Supplementary Table 18. Function enrichment of specific gene families in *O. granulata*.

GO_ID	GO_Term	Adjusted *P*-value
GO:0003676	nucleic acid binding	0
GO:0015074	DNA integration	7.28E-255
GO:0006259	DNA metabolic process	1.65E-234
GO:0097159	organic cyclic compound binding	4.27E-179
GO:1901363	heterocyclic compound binding	4.27E-179
GO:0006139	nucleobase-containing compound metabolic process	8.68E-108
GO:0006725	cellular aromatic compound metabolic process	2.47E-103
GO:0046483	heterocycle metabolic process	1.02E-102
GO:0090304	nucleic acid metabolic process	1.09E-102
GO:1901360	organic cyclic compound metabolic process	1.74E-102
GO:0034641	cellular nitrogen compound metabolic process	1.74E-102
GO:0006807	nitrogen compound metabolic process	7.11E-92
GO:0044237	cellular metabolic process	9.00E-55
GO:0044260	cellular macromolecule metabolic process	2.05E-49
GO:0043170	macromolecule metabolic process	4.28E-40
GO:0022900	electron transport chain	8.35E-40
GO:0015979	photosynthesis	2.86E-36
GO:0005488	binding	5.71E-34
GO:0009767	photosynthetic electron transport chain	7.76E-32
**IPRID**	**IPR Title**	**Adjusted *P*-value**
IPR010811	Domain of unknown function DUF1409	1.20438382486721e-318
IPR019557	Aminotransferase-like, plant mobile domain	2.16E-304
IPR007321	Transposase (putative), gypsy type	1.33E-231
IPR012337	Ribonuclease H-like domain	1.56E-137
IPR000477	Reverse transcriptase	8.55E-75
IPR005162	Retrotransposon gag protein	9.02E-69
IPR001584	Integrase, catalytic core	3.03E-64

Note: here only listed gene families with adjusted *P*-value < 1e-30.

Supplementary Table 19. Function enrichment of bigger gene families in *O. granulata* compared with both of *O. sativa* and *O. brachyantha*.

GO_ID	GO_Term	Adjusted *P*-value
GO:0003676	nucleic acid binding	0
GO:0015074	DNA integration	0
GO:0005488	binding	0
GO:0097159	organic cyclic compound binding	0
GO:1901363	heterocyclic compound binding	0
GO:0006259	DNA metabolic process	0
GO:0090304	nucleic acid metabolic process	2.43E-245
GO:0006139	nucleobase-containing compound metabolic process	7.28E-236
GO:0008270	zinc ion binding	6.99E-234
GO:0006725	cellular aromatic compound metabolic process	4.87E-222
GO:0034641	cellular nitrogen compound metabolic process	1.41E-220
GO:0046483	heterocycle metabolic process	1.41E-220
GO:1901360	organic cyclic compound metabolic process	1.98E-217
GO:0006807	nitrogen compound metabolic process	4.02E-198
GO:0046914	transition metal ion binding	2.62E-153
GO:0046872	metal ion binding	3.99E-84
GO:0043169	cation binding	2.77E-83
GO:0003674	molecular_function	8.37E-74
GO:0046983	protein dimerization activity	3.85E-65
GO:0044260	cellular macromolecule metabolic process	5.74E-51
IPRID	IPR Title	Adjusted *P*-value
IPR000477	Reverse transcriptase	0
IPR005162	Retrotransposon gag protein	0
IPR010811	Domain of unknown function DUF1409	0
IPR012337	Ribonuclease H-like domain	0
IPR019557	Aminotransferase-like, plant mobile domain	0
IPR001584	Integrase, catalytic core	5.43E-280
IPR007321	Transposase (putative), gypsy type	9.44E-280
IPR001878	Zinc finger, CCHC-type	1.78E-172
IPR015401	Transposase, MuDR, N-terminal	7.16E-150
IPR004332	Transposase, MuDR, plant	8.43E-93
IPR009007	Peptidase aspartic, catalytic	1.90E-83
IPR008906	HAT dimerisation	2.75E-83
IPR013242	Retroviral aspartyl protease	3.48E-83
IPR025525	Domain of unknown function DUF4413	9.64E-59

Note: here only listed gene families with adjusted *P*-value < 1e-50.

Supplementary Table 20. Number of Positive Selection Gene (PSG) with single-copy gene.

	Wild		Cultivated		
Species	*O. granulata*	*O. brachyantha*	*O. sativa ssp. indica*	*O. glaberrima*	*O. sativa ssp. japonica*
PSG number	171	170	33	27	30

Step:

(1) 3,746 high-confidence 1:1 orthologous gene families with 7 species;

(2) Get genes under positive selection with codeml (branch site model) in PAML package;

(3) Positive selection results filter FDR threshold (0.05).

Supplementary Table 21. GO function of 171 PSG of *O. granulata*.

Description	Number
GO:0005524: ATP binding function	16
GO:0005515: protein binding function	14
GO:0055114: oxidation-reduction process process	10
GO:0003676: nucleic acid binding function	8
GO:0003824: catalytic activity function	5
GO:0016491: oxidoreductase activity function	5
GO:0006508: proteolysis process	5
GO:0005622: intracellular component	4
GO:0008270: zinc ion binding function	4
GO:0005488: binding function	4
GO:0003735: structural constituent of ribosome function	4

Note: here only listed GO function of 171 PSG >3 genes.

Supplementary Table 22. IPR function of 171 PSG of *O. granulata*.

Description	Number
IPR027417: P-loop containing nucleoside triphosphate hydrolase	12
IPR002885: Pentatricopeptide repeat	5
IPR000504: RNA recognition motif domain	4
IPR017986: WD40-repeat-containing domain	4
IPR001680: WD40 repeat	4
IPR016024: Armadillo-type fold	4

Note: here only listed IPR function of 171 PSG >3 genes.

Supplementary Table 23. Comparison of homolog gene between *O. sativa* and other species.

Query vs Target	Q Number	Q Homolog (%)	T Number	T Homolog (%)	Reciprocal best hits (Q%, T%)	Identity (Mean, Median)
O. sativa vs *A. thaliana*	42,849	24,724 (57.7)	27,331	22,986 (84.10)	10,775 (25.15, 39.42)	49.52, 67.34
O. sativa vs *S. bicolor*	42,849	31,841 (74.31)	34,487	32,868 (95.31)	18,137 (42.33, 52.59)	61.96, 84.00
O. sativa vs *O. nivara*	42,849	32,522 (75.9)	36,245	33,258 (91.76)	28,127 (65.64, 77.60)	84.47, 99.78
O. sativa vs *O. brachyantha*	42,849	32,707 (76.33)	32,023	25,841 (80.70)	19,261 (44.95, 60.15)	68.37, 41.52
O. sativa vs *O. granulata*	42,849	33,195 (77.47)	40,116	33,285 (82.97)	19,564 (45.65, 48.77)	70.41, 90.00

Supplementary Table 24. Statistic analysis of collinearity between *O. sativa* and other species.

Query vs Target	Syntenic blocks	Average syntenic gene pairs per block	Syntenic gene pairs	Mean block length	Total Q gene (%) of syntenic blocks	Total T gene (%) of syntenic blocks
O. sativa vs *A. thaliana*	391	8.4578	3,307	169268.1714/361110.3146	2,528 (4.52)	2,538 (9.29)
O. sativa vs *S. bicolor*	3029	14.1466	42,850	511382.0376/3980560.8174	22,506 (40.21)	19,007 (55.11)
O. sativa vs *O. nivara*	1004	30.5568	30,679	523270.4572/553316.9293	25,363 (45.32)	25,093 (69.23)
O. sativa vs *O. brachyantha*	609	41.3875	25,205	921280.5484/854799.4631	19,763 (35.31)	19,083 (59.59)
O. sativa vs *O. granulata*	1597	12.469	19,913	220172.9480/279211.8359	16,875 (30.15)	16,123 (40.19)
