# Supplementary material for: De novo genome assembly of Oryza granulata reveals rapid genome expansion and adaptive evolution
Source: Commun Biol. 2018 Jun 29;1:84. doi: 10.1038/s42003-018-0089-4 (PMC6123737; doi:10.1038/s42003-018-0089-4)
Supplement: Supplementary file 2 — Description of additional supplementary items [file 42003_2018_89_MOESM2_ESM.docx]

**Description of Additional Supplementary Files**

File Name: Supplementary Data 1

Description: Inserting traces of LTRs in the centromere-specific contig2720

File Name: Supplementary Data 2

Description: Function enrichment of expanded gene families in *O. granulate* and contracted in *O. sativa*

File Name: Supplementary Data 3

Description: Comparison GO among *O. granulata*, *O. sativa* and *O. brachyantha*

File Name: Supplementary Data 4

Description: GO function of significantly expanded gene families in *O. granulata*

File Name: Supplementary Data 5

Description: GO Enrichment of 171 PSG of *O. granulata*

File Name: Supplementary Data 6

Description: IPR Enrichment of 171 PSG of *O. granulata*
